# Myosin Va-dependent Transport of NMDA Receptors in Hippocampal Neurons

**DOI:** 10.1007/s12264-023-01174-y

**Published:** 2024-01-30

**Authors:** Ru Gong, Linwei Qin, Linlin Chen, Ning Wang, Yifei Bao, Wei Lu

**Affiliations:** 1 Ministry of Education (MOE) Key Laboratory of Developmental Genes and Human Disease, School of Life Science and Technology, Southeast University, Nanjing, 210096 China; 2 Department of Neurosurgery, State Key Laboratory of Medical Neurobiology, MOE Frontiers Center for Brain Science, Huashan Hospital, Institute for Translational Brain Research, Fudan University, Shanghai, 200032 China; 3 Department of Neurobiology, Nanjing Medical University, Nanjing, 210096 China; 4 Co-innovation Center of Neuroregeneration, Nantong University, Nantong, 226001 China

**Keywords:** Myosin Va, NMDA receptor, CaMKII, Transport, Memory

## Abstract

N-methyl-D-aspartate receptor (NMDAR) trafficking is a key process in the regulation of synaptic efficacy and brain function. However, the molecular mechanism underlying the surface transport of NMDARs is largely unknown. Here we identified myosin Va (MyoVa) as the specific motor protein that traffics NMDARs in hippocampal neurons. We found that MyoVa associates with NMDARs through its cargo binding domain. This association was increased during NMDAR surface transport. Knockdown of MyoVa suppressed NMDAR transport. We further demonstrated that Ca^2+^/calmodulin-dependent protein kinase II (CaMKII) regulates NMDAR transport through its direct interaction with MyoVa. Furthermore, MyoVa employed Rab11 family-interacting protein 3 (Rab11/FIP3) as the adaptor proteins to couple themselves with NMDARs during their transport. Accordingly, the knockdown of *FIP3* impairs hippocampal memory. Together, we conclude that in hippocampal neurons, MyoVa conducts active transport of NMDARs in a CaMKII-dependent manner.

**Supplementary Information:**

The online version contains supplementary material available at 10.1007/s12264-023-01174-y.

## Introduction

The N-methyl-D-aspartate receptor (NMDAR) is a subtype of glutamate receptor important for postsynaptic response modulation and thus plays a crucial role in various brain functions [1]. The number of synaptic NMDAR components can be altered in response to electrophysiological inputs or sensory cues [1–9]. Transport of new NMDARs into dendritic spines increases the number of postsynaptic NMDARs, thereby facilitating the subsequent occurrence of synaptic plasticity and memory consolidation [1, 7, 10–13]. In another light, dysregulated NMDAR transport has been found in the pathophysiology of neural disorders such as Alzheimer’s disease and schizophrenia [10, 14, 15]. In contrast to extensive studies on the role of AMPAR (α-amino-3-hydroxy-5-methyl-4-isoxazolepropionic acid receptor) trafficking in the plasticity of AMPAR-mediated synaptic responses, only a few studies have documented the requirement of NMDAR transport for NMDAR-mediated synaptic plasticity.

After being released from the endoplasmic reticulum, the assembled NMDARs are sent *via* Golgi bodies to the neuronal surface [16, 17]. The journey from the soma to the dendritic spine requires members of the kinesin motor protein family, together with adaptor proteins, to transport NMDARs along microtubules in dendrites. Kinesin family 3B (KIF3B) and KIF17 are respectively responsible for the transport of NMDARs containing GluN2A and GluN2B subunits [10, 11, 13, 18]. Once they arrive at the base of the dendritic spine, NMDARs are believed to be released from KIF and transferred to myosin proteins that are associated with F-actin [19–21], which provides cytoskeletal support in dendritic spines.

Among the great family of motor proteins, unconventional class V myosins have been found to be associated with the active transport of diverse cargos along actin filaments [20, 22, 23], which makes them attractive candidates for the transport of synaptic proteins. Of the three class V myosins, only myosin Va (MyoVa) and myosin Vb (MyoVb) are present in the brain and possess features that allow them to serve as effective organelle motors within neurons [20, 22]. To date, it is unknown which type of myosin conducts NMDAR transport in spines. Considering the crucial role of NMDAR trafficking in both physiological brain functions and various neurological disorders, it is of great importance to uncover the specific motor protein and the process of regulation of NMDAR trafficking.

In this study, we specified that MyoVa, but not MyoVb, associates with NMDARs through its globular, cargo-binding domain in CA1 hippocampal neurons. This bond is fortified during NMDAR trafficking. Using a combination of biochemical, immunofluorescent, and electrophysiological measurements, we further demonstrated that MyoVa conducts a Ca2+/calmodulin-dependent protein kinase II (CaMKII)-dependent surface transport of NMDARs. This transport process requires Rab11 family-interacting protein 3 (Rab11/FIP3) as the adaptor proteins to couple NMDARs and MyoVa. Taken together, these results support MyoVa as the specific motor protein that conducts NMDAR transport, which is important for the formation of hippocampal memory.

## Materials and Methods

### Animals

All animal-related procedures were approved by the Experimental Animal Ethics Committee at Southeast University. Male and female Sprague−Dawley (Charles River, Beijing, China) rats were housed in a temperature-controlled (26°C) vivarium and maintained on a 12-h/12-h light/dark cycle (lights on from 07:00 to 19:00) with *ad libitum* access to food and water. Male and female rats, 14–16 days old, were used in electrophysiology and Western blotting. One-month-old male rats were used in behavioral testing experiments.

### Peptides

The interfering peptide Tat-MyoVa (4 μmol/L, YGRKKRRQRRR-PKPGHKRTDSTHSSNESEY) was designed based on the CaMKII binding sequence in MyoVa. The sequence of the scrambled peptides was YGRKKRRQRRR-YHSPSDGKSNTSHPTKERE, in which YGRKKRRQRRR represents the structural domain that mediates cell membrane conductance. Peptides were synthesized by ChinaPeptides Co. (Nanjing, China) and applied in artificial cerebrospinal fluid (ACSF) used in patch-clamp recording 20 min prior to the induction of long-term potentiation (LTP).

### Virus

A sequence corresponding to 5344–5362 nucleotides (5’-GGT CTC TGT TTC ATT TAT C-3’) in rat *MyoVa* messenger RNA was used to inhibit endogenous rat MyoVa. A nonspecific scrambled short-hairpin RNA (shRNA) was treated as a control (5’-GGT TTC GTA CTT TCT CTT A-3’). The sequences were packaged into rAAV2/9 (Shanghai Sunbio Medical Biotechnology, Shanghai, China) or lentiviral particles (BrainVTA, Wuhan, China) tagged with mCherry. The flag-tagged wild-type (rescue) and 1725 mutant (Q1725A mut, the 1725 site is homologous to 1750 in humans) *MyoVa* gene resistant to knockdown (KD) were packaged into lentiviral particles by BrainVTA. The nucleotides used for endogenous rat *FIP3* KD (5’-GCA TTC TGC TAC TTG CTA AAG-3’), and the nonspecific scrambled shRNA (5’-CGC TGA GTA CTT CGA AAT GTC-3’) were tagged with the enhanced green fluorescent protein (*EGFP*) and packaged into rAAV2/9 by Shanghai GeneChem (Shanghai, China).

### *In Vivo *Stereotactic Injections

One-week-old rats used in electrophysiology experiments and 8-week-old rats used in behavior tests were prepared for stereotaxic injection [24, 25]. Rats were anesthetized with isoflurane and then immobilized on a stereotaxic apparatus. Small bilateral holes were drilled into the skull and then concentrated virus solution (rAAV2/9 expressing *MyoVa* or *FIP3* KD shRNA) was injected either unilaterally into the CA1 region of 1-week-old (300 nL, bregma −2.0 mm; lateral ±2.5 mm; ventral ±2.4 mm) or bilaterally into 8-week-old rats (400 nL, bregma −3.5 mm; lateral ±2.8 mm; ventral ±2.6 mm) with a glass pipette attached to a microinjection pump (World Precision Instruments WPI, Sarasota, USA). After the virus injection, the needle was left in place for 10 additional min. Two weeks after the AAV injection, the rats were sacrificed for extracellular recordings, Western blot, or behavioral tests.

To verify virus expression in the CA1 region of the hippocampus, stereotaxic slices were obtained 2 weeks after virus injection. Rats were anesthetized with isoflurane and cardio-perfused. The brain was removed and fixed in 4% paraformaldehyde for 24 h and soaked in 40% sucrose for 2–3 days for dehydration. The 35-μm-thick hippocampal sections of the CA1 region were cut on a microtome cryostat (CM1950, Leica, Wetzlar, Germany) and then cover-slipped with mounting medium (Southern Biotech, Birmingham, USA) containing DAPI. Images were captured under an upright microscope (DM5000B, Leica) or laser scanning confocal microscope (LSM700, Zeiss, Oberkochen, Germany).

### Acute Hippocampal Slice Preparation

Rats were anesthetized with isoflurane and rapidly decapitated. The isolated brain was quickly placed in cold ACSF containing (in mmol/L): 124 NaCl, 5 KCl, 1.2 KH_2_PO_4_, 1.3 MgSO_4_, 2.4 CaCl_2_, 26 NaHCO_3_, and 10 glucose (pH 7.4). Then the hippocampal tissue was cut into 350 μm thick slices on a Leica vibratome. After incubation at 34°C for 1 h in oxygenated ACSF continuously ventilated with 95% O_2_ and 5% CO_2_, the slices were acclimated to room temperature for another 30 min. During recordings, slices were placed in a chamber filled with ACSF continuously ventilated with 95% O_2_ and 5% CO_2_.

### Electrophysiology

Patch pipettes were made with a horizontal microelectrode puller (P-1000, Sutter Instruments, Novato, USA). The average resistance was 3–5 MΩ. To evoke field excitatory postsynaptic potentials (fEPSPs) in CA1, a bipolar stimulating electrode (FHC, Bowdoin, USA) was placed in the stratum radiatum to stimulate Schaffer commissural projections at 0.05 Hz. The resistance of the glass electrode filled with 2 mmol/L NaCl to record fEPSP was 3–5 MΩ. NMDAR-mediated fEPSPs were recorded in ACSF with a low Mg^2+^ (0.25 mmol/L) concentration. 10 μmol/L NBQX (2,3-dihydroxy-6-nitro-7-sulfamoylbenzo quinoxaline-2,3-dione) was added to the recording solution to block AMPAR-mediated excitatory synaptic currents (EPSCs). The EPSCs of NMDAR were obtained by whole-cell recording with a patch pipette (4–6 MΩ) containing (in mmol/L) 135 K-gluconate, 5 KCl, 2 NaCl, 0.2 EGTA, 10 HEPES, 5 ATP, and 5 QX-314 (pH 7.4). Cells were held at −65 mV in the low concentration of Mg^2+^ (0.25 mmol/L) ACSF. 10 μmol/L BMI (bicuculline methiodide) and 10 μmol/L NBQX were applied in the recording solution for the blockade of GABA_A_ and AMPA receptors, respectively. NMDAR-mediated miniature EPSCs (mEPSCs) were recorded at −65 mV in Mg^2+^-free ACSF containing NBQX (10 μmol/L), BMI (5 μmol/L), and tetrodotoxin (TTX, 1 μmol/L). NMDAR-dependent LTP was induced by theta-burst stimulation (TBS, 3 times 5 bursts of 5 Hz, each burst containing 5 stimuli at 100 Hz, with a 30 s interburst interval, for references see [26]) or incubation with the protein kinase C (PKC) agonists PMA (phorbol myristate acetate, 0.5 μmol/L, for references see [27–29]) for 10 min. NMDAR-dependent LTD (long-term depression) was induced by low-frequency stimulation containing 900 stimuli at 1 Hz [30]. Individual experiments were normalized to the baseline to generate a summary graph, and three consecutive reactions were averaged for 1-min plots. The magnitudes of LTP or LTD were calculated based on the averaged fEPSP or EPSC values in the last 10 min of recording after induction. Clampex 10.4 and Clampfit 10.4 (Axon Instruments, San Jose, USA) were used for data recording and analysis, respectively.

### Primary Hippocampal Cell Culture

Primary hippocampal cultures were prepared from E18–19 rats as described previously [31, 32]. Hippocampi were dissected and digested with 0.125% trypsin (Gibco, CA, USA) diluted in FBS (fetal bovine serum) for 8 min at 37°C, then the reaction was quenched with 10% FBS (Gibco) diluted by Dulbecco’s modified Eagle’s medium. Neurons were plated on coverslips coated with 0.1 mg/mL poly-D-lysine (Gibco) at a density of 50,000–80,000 cells per cm^2^ (for imaging studies) or 300,000 cells per cm^2^ (for Western blots). Neurons were grown in Neurobasal medium (Gibco) supplemented with B-27 (Gibco) containing glutamine (Gibco), β-mercaptoethanol (Gibco), and 1× penicillin-streptomycin (Gibco). Cultures were kept in a humid 5% CO_2_ tissue culture incubator at 37°C. Half fresh medium was exchanged every 3 days until experiments. Neurons were infected with *MyoVa*-shRNA or *MyoVa* scrambled-shRNA at 7 days *in vitro* (DIV) by adding rAAV2/9-packaged viruses into wells with the final virus titer at 10^9^–10^10^ genome copies/mL. For the expression of MyoVa or Q1725A mutant MyoVa in neurons, lentivirus was added at DIV7.

### Western Blotting

The hippocampal slices were homogenized in cold homogenization buffer (pH 7.4) containing (in mmol/L) 320 sucrose, 1 HEPES, 1 MgCl_2_, 1 NaHCO_3_, 20 sodium pyrophosphate, 20 β-phosphoglycerol, 0.2 dithiothreitol, 1 EDTA, 1 EGTA, 50 NaF, and 1 Na_3_VO_4_. Protease inhibitors and phosphatase inhibitors were also added, which included 1 mmol/L phenyl-methylsulfonyl fluoride (PMSF), 1 mmol/L p-nitrophenyl phosphate, 5 µg/mL aprotinin, 5 µg/mL leupeptin, 5 µg/mL pepstatin A, and 16 µg/mL benzamidine. The sample was centrifuged at 1,000× *g* for 10 min. The supernatant was used for total protein analysis. To obtain the Triton insoluble fraction (TIF) [33, 34], the supernatant was centrifuged at 3,000× *g* for 15 min and the resulting pellet was resuspended in 8 mL of hypotonic buffer with protease inhibitors, followed by centrifugation at 100,000× *g* for 1 h. The resulting pellet was resuspended and centrifuged for 1 h at 100,000× *g* in 8 mL of buffer containing 75 mmol/L KCl and 1% Triton X-100. The final pellet was homogenized three times in 20 mmol/L HEPES, and the resulting fraction was referred to as the TIF. The concentration of total or TIF proteins was determined with the Bradford protein assay kit (Thermo Fisher, Waltham, USA). Equal amounts of proteins were denatured by 5× SDS (sodium dodecyl sulfate)-loading buffer and then separated by SDS-PAGE using precast 8% gels. Proteins on the gels were transferred to PVDF (hydrophobic polyvinylidene fluoride) membranes (BioRad, Hercules, USA) and blocked in 3% BSA (bovine serum albumin) in TBST solution for 2 h at room temperature. Then, the membranes were incubated with primary antibodies diluted in 1% BSA in TBST solution at 4°C overnight. After washing 5 times in TBST, the membranes were incubated with the corresponding secondary antibody diluted in 1% BSA in TBST solution for 1 h at room temperature. The membranes were then washed another 5 times. Results were visualized using an imaging system (Tanon, Shanghai, China) and analyzed by ImageJ. The major primary antibodies were: MyoVa (Sigma, St. Louis, USA, HPA001356), GluN2A (Millipore, Burlington, USA, AB1555P), GluN1 (rabbit: Abclonal, Wuhan, China, A11699; mouse: Abcam, Cambridge, UK, ab134308), GluA1 (Millipore, MAB2263), GluN2B (Cell Signaling Technology, Boston, USA, 14544), Rab11 (Invitrogen, Carlsbad, USA, 71-5300), FIP3 (Rockland, Philadelphia, USA, 600-401-994), PSD95 (Millipore, MABN68), MyoVb (Sigma, HPA040902), CaMKII (Santa Cruz Biotechnology, Inc., Dallas, USA, sc-13141), GST (glutathione-S-transferase, Genscript, Nanjing, China, A00866-100). The secondary antibodies were: goat anti-rabbit IgG-HRP (horseradish peroxidase, Thermo Fisher Scientific, Waltham, USA, A16110) and goat anti-mouse IgG-HRP (Jackson ImmunoResearch, West Grove, USA, 115-035-003). For visualization of the signal, the Super Signal West Pico Chemiluminescent Substrate Kit (Thermo Fisher Scientific, 34580) was used according to the manufacturer’s instructions.

### Co-immunoprecipitation (Co-IP)

Total hippocampal lysates were incubated with primary antibodies in the immunoprecipitation buffer at 4°C by end-over-end spinning. The immunoprecipitation buffer (pH 7.4) contained 0.05 mol/L HEPES, 0.15 mol/L NaCl, 10% glycerol, 0.5% Nonidet P-40, 1% TritonX-100, 1 mmol/L EGTA, 1 mmol/L EDTA, 1 mmol/L Na_3_VO_4_, and 1 mmol/L PMSF. After equilibration, the Protein A/G agarose (Thermo Fisher Scientific) was pipetted into the solution and mixed at 4°C overnight. After 5 washes with 1% TritonX-100 buffer, the bound proteins were eluted by boiling with 2× SDS buffer for 8 min, separated by SDS-PAGE, and analyzed by immunoblotting.

### Glutathione S-transferase (GST) Pull-down Assays

The GST constructs of the rat MyoVa globular tails (amino acids 1,430–1,830) and medial tail (amino acids 1,110–1,440) were generated by Genscript as described in previous studies (Correia *et al*., 2008; Costa *et al.*, 1999). Equal amounts of total proteins from hippocampal slices or cells were mixed with glutathione resin (Genscript), together with either GST-MyoVa globular tails, medial tail sequence fusion proteins, or GST alone at 4°C overnight *via* end-over-end spinning. The beads were processed by centrifugation and washing 5 times with PBS (phosphate-buffered saline) containing 0.1% Triton X-100. The conjugated proteins were eluted by boiling in 2× SDS sample buffer for 10 min, separated by SDS-PAGE, and analyzed by immunoblotting.

### Surface Biotinylation Assay

The surface biotinylation assay was conducted to detect the expression of NMDAR proteins on the plasma membrane, as described in the Pierce Cell Surface Protein Biotinylation and Isolation Kit (Pierce, Thermo Fisher). At DIV16, hippocampal neurons infected with the virus were treated with or without PMA for 10 min and then were incubated with 1 mg/mL EZ-Link™ Sulfo-NHS-SS-Biotin at 4°C for 45 min. After the labeling solution was removed from the wells, neurons were washed three times with ice-cold TBS and harvested by gentle scraping. Neurons were then transferred into the lysis buffer containing protease inhibitors and centrifuged at 15,000× *g* for 10 min at 4°C. Supernatants containing equal amounts of protein were incubated with NeutrAvidin™ agarose in a column overnight at 4°C to capture biotinylated (surface) proteins *via* an end-over-end mixing rotator. After 5 washes with the wash buffer in the kit, the captured surface proteins were eluted from the biotin-NeutrAvidin agarose by incubation with dithiothreitol (10 mmol/L) in elution buffer for 30 min at room temperature on an end-over-end rotator. The eluted proteins, representing the cell surface proteins, were collected by column centrifugation. The samples were then separated using SDS-PAGE and imaged by Western blotting.

### Immunofluorescence Labeling and Analysis

Immunofluorescence was used to analyze NMDAR trafficking in primary hippocampal cultures as previously described [31, 35]. The GluN1 expressed on the surface of the neuronal plasma membrane or in the intracellular compartment were labeled with the corresponding antibodies under membrane-impermeable or membrane-permeable conditions, respectively. Surface GluN1 was labeled by incubating hippocampal neurons with mouse GluN1 antibodies (Abcam) for 40 min at room temperature, followed by 5 washes with PBS, fixation in ice-cold parafix solution (4% paraformaldehyde, 4% sucrose in PBS) for 15 min, and 5 washes with PBS. Neurons were then incubated with Alexa fluor 488-conjugated secondary antibody (Invitrogen) for 1 h at room temperature. Following permeabilization (0.3% Triton X-100 in PBS) and blocking (10% normal donkey serum, 1 h) at room temperature, total GluN1 was labeled with rabbit GluN1 antibodies (ABclonal) overnight at 4°C. After incubation with Alexa fluor 647 secondary antibodies (Abcam) for 1 h at room temperature and 5 washes with PBS, sections were then mounted on glass slides coverslipped with Fluoromount-G (SouthernBiotech). A 63× oil-immersion objective on a Zeiss LSM900 confocal microscope was used to capture images. Consecutive optical sections were collected at 0.35 μm intervals to create the maximum projection image. Fluorescence intensities from 3 independent areas per neuron were measured using Fiji software (National Institutes of Health, West Grove, USA) for surface (Alexa fluor 488-conjugated secondary antibody) and total GluN1 (Alexa fluor 647 secondary antibodies). Data are presented as surface/total GluN1 ratios.

For co-localization analysis of GluN1 and MyoVa (GluN1/MyoVa), GluN1/PSD95, GluN1/FIP3, or CaMKII/MyoVa, hippocampal neurons at DIV 16–18 were fixed and permeabilized as above and then blocked with 10% normal donkey serum for 1 h at room temperature. Primary antibodies labeling pairs of proteins (GluN1/MyoVa, GluN1/PSD95, GluN1/FIP3, or CaMKII/MyoVa) were incubated overnight at 4°C. Following 5 washes, corresponding secondary antibodies were incubated for 2 h at room temperature. After 5 washes, the coverslips were mounted on glass slides. Images were acquired with a 63 × oil immersion objective on a Zeiss LSM900 confocal microscope under the same exposure, gain, and intensity conditions and analyzed with Fiji software (National Institutes of Health) using the same parameters.

### Behavioral Tests

#### Fear Conditioning

Before the beginning of the experiments, rats were handled for 5 days. The training and test procedures for both contextual and trace fear conditioning were performed as previously described [36]. On the first day, the rats were acclimatized for 12 min in a training room with metal walls and a stainless-steel grid floor. On day 2, rats were allowed to explore the chamber for 3 min before the onset of six consecutive training blocks, each consisting of a 20 s baseline, a 20 s, 2 kHz, 80 dB tone (conditioned stimulus), an 18 s trace interval of silence, followed by a 0.8 mA foot shock persisting for 2 s (unconditioned stimulus) and lastly an intertrial interval (ITI) persisting for 40 s. Memory tests were applied on the third day. For contextual memory recall, rats were first placed in the training chamber for 3 min to assess contextual fear responses. For tone-cued memory recall, rats were placed in a novel chamber with walls and floors made of acrylic panels, in which they were allowed free exploration for 3 min and then presented with four testing blocks, each consisting of a 20 s baseline, a tone (20 s, 2 KHz, 80 dB), and a 60 s ITI. The chamber was cleaned with 10% ethanol after each rat was tested. The Freeze Frame (Actimetrics, Wilmette, USA) software for automatic motion detection was used to quantify the percentage of time the animal spent in freezing behaviors.

#### Novel Object Recognition (NOR) Task

Rats were handled for 5 days and habituated to an open-field box (100 cm × 100 cm × 35 cm) for 3 days. The training and test procedures were executed as described [37]. Rats were allowed to explore two novel objects for 10 min during training sessions on the first day, and the time for rats to explore each object was recorded. In the test sessions on day 2, rats were positioned in the same box to explore two objects for 10 min, one of which was the familiar object, and the other was replaced by a novel object. To avoid the influence of olfactory cues, the objects were thoroughly cleaned with 10% ethanol after exploration by the rats. Recognition memory was measured by a preference index using Anymaze (Stoelting, Wood Dale, USA), the ratio of the time spent exploring either of the two objects (training session) or the new object (test session) to the total time spent exploring the two objects.

#### Novel Place Preference (NPP) Task

Rats were handled and habituated as described above before training. During the training phase, rats were placed in the open-field box to explore two identical objects located in the corners of the box for 10 min. On day 2, one copy of the objects was placed at a different location, whereas the other one was placed in the same location as during the training trials. Recognition memory was measured by taking the ratio of the time spent exploring the relocated object to the total time spent exploring both objects using Anymaze.

#### Temporal Order Memory Task

Rats were handled and habituated as described above before training. Experiments were performed following protocols previously described [38]. Rats were placed in an experimental apparatus to explore each set of three objects (referred to as A–A, B–B, and C–C) for 10 min with a 3-min inter-session interval. The rats were given a 3-min timeout after the third set of objects and then were returned to the apparatus where a third copy of object A and a third copy of object C were placed at opposite ends of the box. The preference for object A *versus* object C in a 10-min period was measured by Anymaze (Stoelting). The overall activity level for the temporal order task was calculated as the ratio of the time spent exploring object A to the total time spent exploring objects A and C.

#### Open-field Tests

Open-field tests were used to test general behavior and locomotor activity as previously described [39]. Rats were paced in an open-field box (100 cm × 100 cm × 35 cm) and left to freely move for 3 min. Between tests for each rat, the test chamber was cleaned with 10% ethanol. Motion trails were recorded by video tracking through which the total distance, mean speed, and time mobile were measured and analyzed using the Anymaze system.

### Quantification and Statistical Analysis

Data in the bar graphs are presented as the mean ± SEM and statistical analysis of the data was calculated by GraphPad software. Student’s *t*-test was applied to compare differences between the two groups. One-way ANOVA (analysis of variance) was applied to compare the differences between more than two groups. Two-way repeated measures ANOVA was used to assess how data from different groups evolved over time, that is, how animal freezing rates changed over time. The association between the two variables was evaluated by Pearson’s correlation coefficient. When *P* <0.05 (*P <0.05, **P <0.01), the difference was considered significant.

## Results

### Increased Association Between MyoVa and NMDARs During NMDAR Transport

**Fig. 1:**
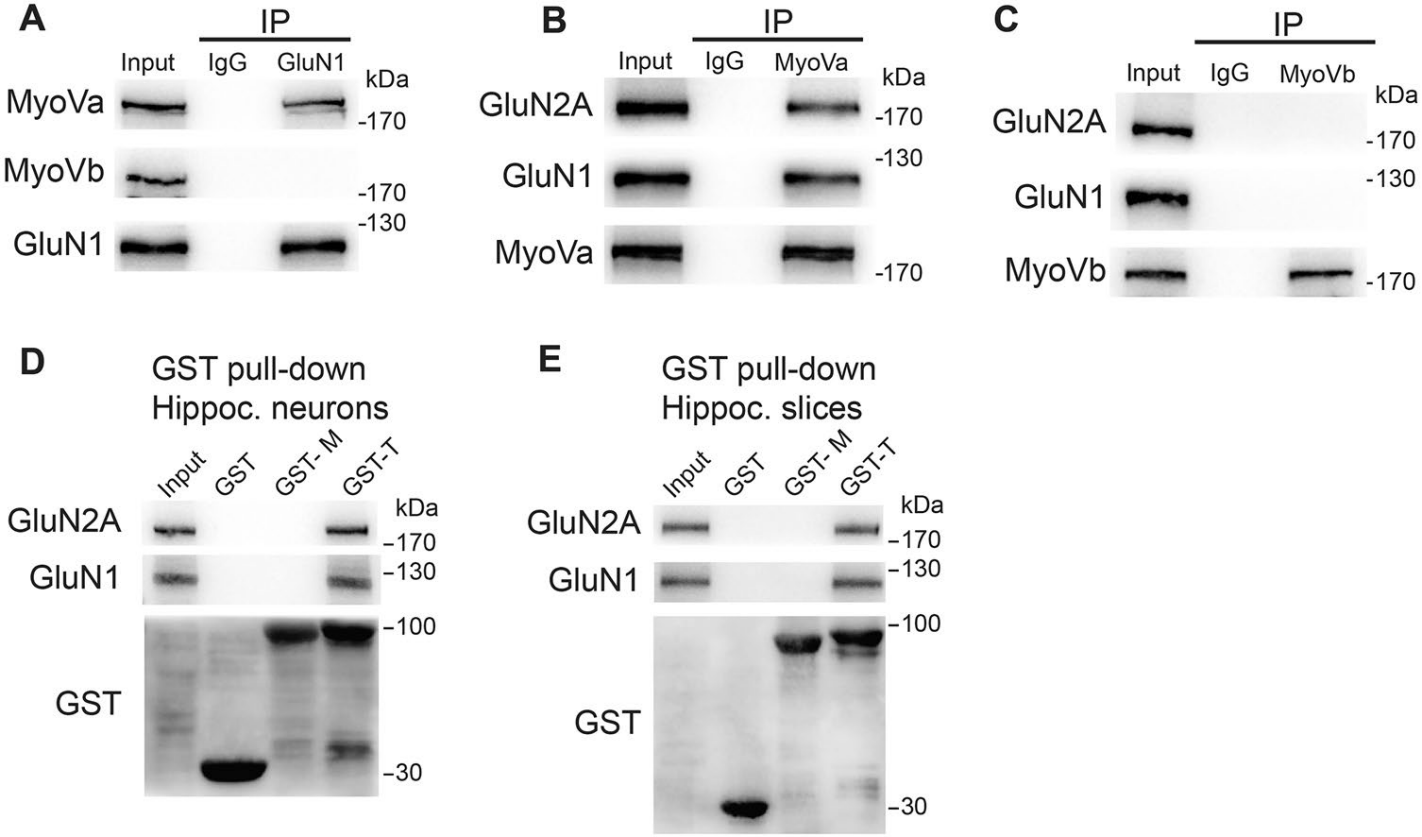
MyoVa associates with NMDARs *via* its globular, cargo-binding domain. **A–C** Co-immunoprecipitation (co-IP) of rat hippocampus homogenates with antibodies against GluN1 (**A**), MyoVa (**B**), or MyoVb (**C**). IgG is reported as control and immunoblotted (IB) with the indicated antibodies. Results were confirmed by at least three independent experiments. **D–E** GST pull-down assays. The medial tail (GST-M) or globular tail (GST-G) of MyoVa fused to GST was incubated with homogenates of hippocampal (Hippoc.) neurons (**D**) or hippocampal slices (**E**). Western blot showing that GluN1 and GluN2A subunits combine with the globular tail domain (GTD) of MyoVa (GST-G) but not the medial tail of MyoVa (GST-M). Plain GST was used as a control. Results were confirmed by at least three independent experiments.

To investigate whether MyoVa or MyoVb is involved in the transport of NMDARs, we first examined the possible association between MyoVa or MyoVb with NMDARs. We performed standard co-IP experiments on hippocampal homogenates with antibodies to GluN1, MyoVa, and MyoVb. As shown in Fig. 1A, MyoVa, but not MyoVb, was specifically immunoprecipitated by an anti-GluN1 antibody. Consistent with this, GluN1 and GluN2A were specifically immunoprecipitated by MyoVa (Fig. 1B), but not by MyoVb antibody (Fig. 1C). This selectivity for myosin association also applied to GluN2B (Fig. S1A).

To confirm the association between MyoVa and NMDARs, we used GST pull-downs targeting different domains of the MyoVa C terminus in both cultured hippocampal neurons (Fig. 1D) and hippocampal slices (Fig. 1E). We found that the globular tail of MyoVa (cargo-binding domain) was associated with both GluN1 and GluN2A NMDAR subunits in hippocampal neurons, whereas the plain GST did not. These results indicate that MyoVa associates with NMDARs in hippocampal neurons under basal conditions.

**Fig. 2:**
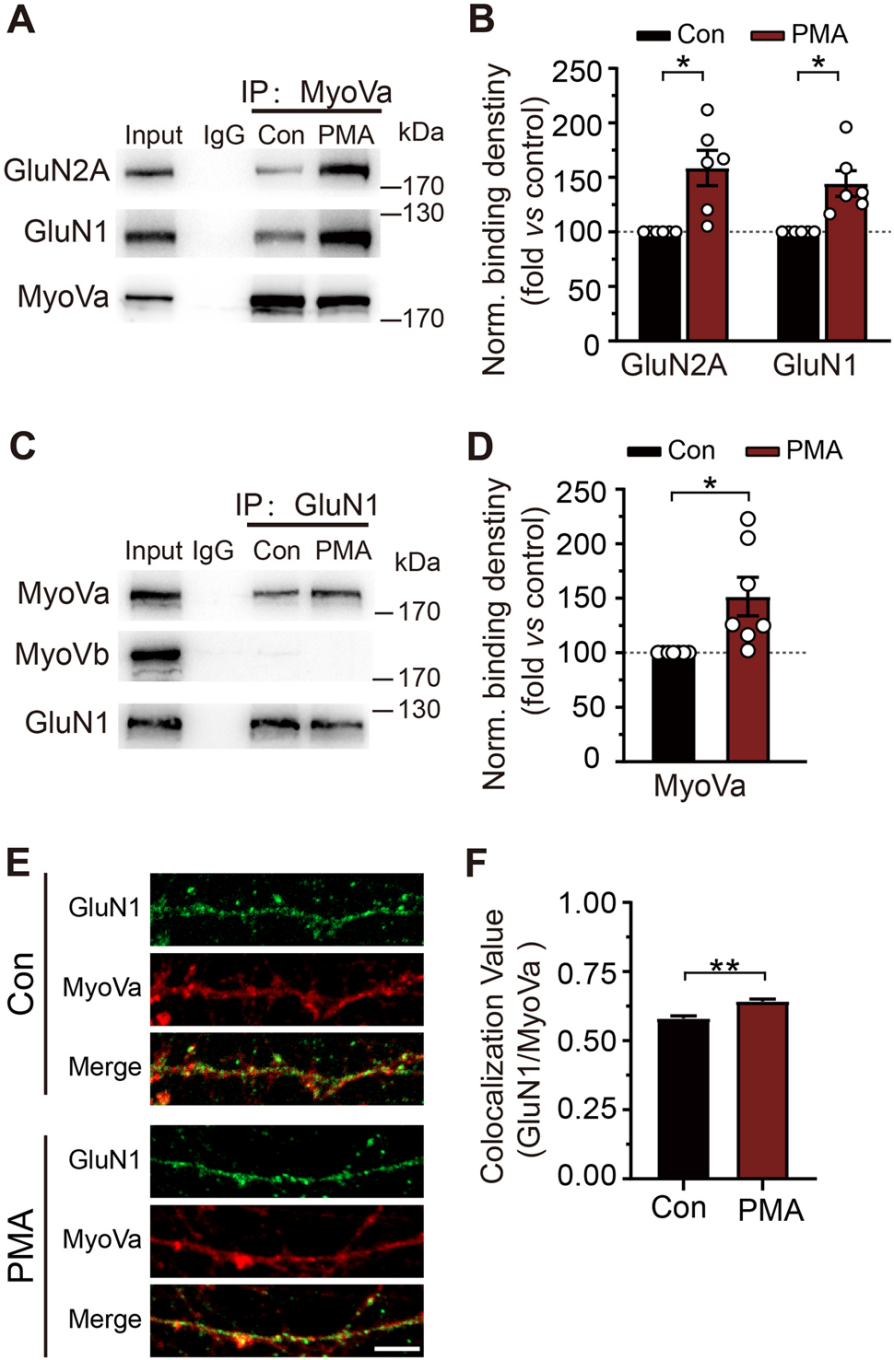
MyoVa-NMDAR association is enhanced during PMA-induced NMDAR transport. **A** Co-IP between MyoVa and associated proteins with antibodies to MyoVa in control (Con) or with PMA treatment. Non-immune IgG was used as control. **B** Statistical analysis of binding between MyoVa and associated proteins. NMDAR signals were divided by MyoVa signals and normalized to control. For GluN2A, the binding increased to 1.59 ± 0.16, *P* <0.05; for GluN1, the binding increased to 1.44 ± 0.12, *P* <0.05; *n* = 6; unpaired Student’s *t*-test *versus* control. **C** Co-IP between MyoVa and GluN1 with antibodies to GluN1 in control or with PMA. Non-immune IgG was used as control. **D** Statistical analysis of binding between MyoVa and GluN1. MyoVa signals were divided by GluN1 signals and normalized to control. The binding increased to 1.52 ± 0.18, *P* <0.05; *n* = 7; unpaired Student’s *t*-test *vs* control. **E** Representative images of primary hippocampal neurons stained with GluN1 (green) and MyoVa (red) antibodies in control or with PMA. Scale bar, 5 μm. **F** Quantification of the colocalization between GluN1 and MyoVa by Pearson’s coefficient. Control: *n* = 59 neurons, 0.58 ± 0.01; PMA: *n* = 71 neurons, 0.61 ± 0.01, *P* <0.01, data from at least three independent cultures; unpaired Student’s *t*-test *vs* control. The data are represented as the mean ± SEM, ^*^P <0.05; ^**^P <0.01.

Activation of PKC rapidly transports NMDARs to the cell surface by phosphorylating GluN1 [29, 40, 41]. To further examine a possible alteration in the MyoVa-NMDAR association during NMDAR transport, we carried out co-IP analyses on hippocampal homogenates upon activation of PKC by phorbol myristate acetate (PMA, 0.5 μmol/L). Consistent with the previous studies [27, 28], perfusing brain slices with PMA for 10 min elicited enhancement of the surface expression of NMDARs at the postsynaptic site (Fig. S1B, C). As a control, the total amount of protein expression was not altered (Fig. S1D, E). More importantly, MyoVa showed a dramatic enhancement in its association with NMDARs, revealed by experiments with antibodies to MyoVa (GluN2A, 1.59 ± 0.16, *P* <0.05; GluN1, 1.44 ± 0.12, *P* <0.05; *n* = 6; Fig. 2A, B) and GluN1 (MyoVa, 1.52 ± 0.18, *P* <0.05; *n* = 7; Fig. 2C, D). By contrast, no NMDAR association with MyoVb was detected after PKC activation, reiterating the involvement of MyoVa rather than MyoVb in NMDAR transport (Fig. 2C). Moreover, we failed to detect any association between MyoVa and GABA receptors under control or PMA conditions (Fig. S1F).

To further confirm the enhancement in MyoVa-NMDAR association during NMDAR transport, we performed immunolabeling analyses of the endogenous proteins in dendrites from cultured hippocampal neurons. As shown in Fig. 2E, MyoVa displayed a partial colocalization with GluN1 under basal conditions. PMA treatment substantially enhanced the colocalization value (control: *n* = 59 neurons, 0.58 ± 0.01; PMA: *n* = 71 neurons, 0.61 ± 0.01, *P* <0.01; Fig. 2E, F). Consistent with the co-IP experiment (Figs. 1A, C and 2C), the Pearson coefficient for MyoVb/GluN1 was significantly lower than MyoVa/GluN1, indicating that GluN1 increased its association with MyoVa rather than with MyoVb (Fig. S1G, H). Taken together, these data indicate that MyoVa increases its association with NMDARs during NMDAR transport.

### *MyoVa *KD Affects NMDAR Transport

**Fig. 3:**
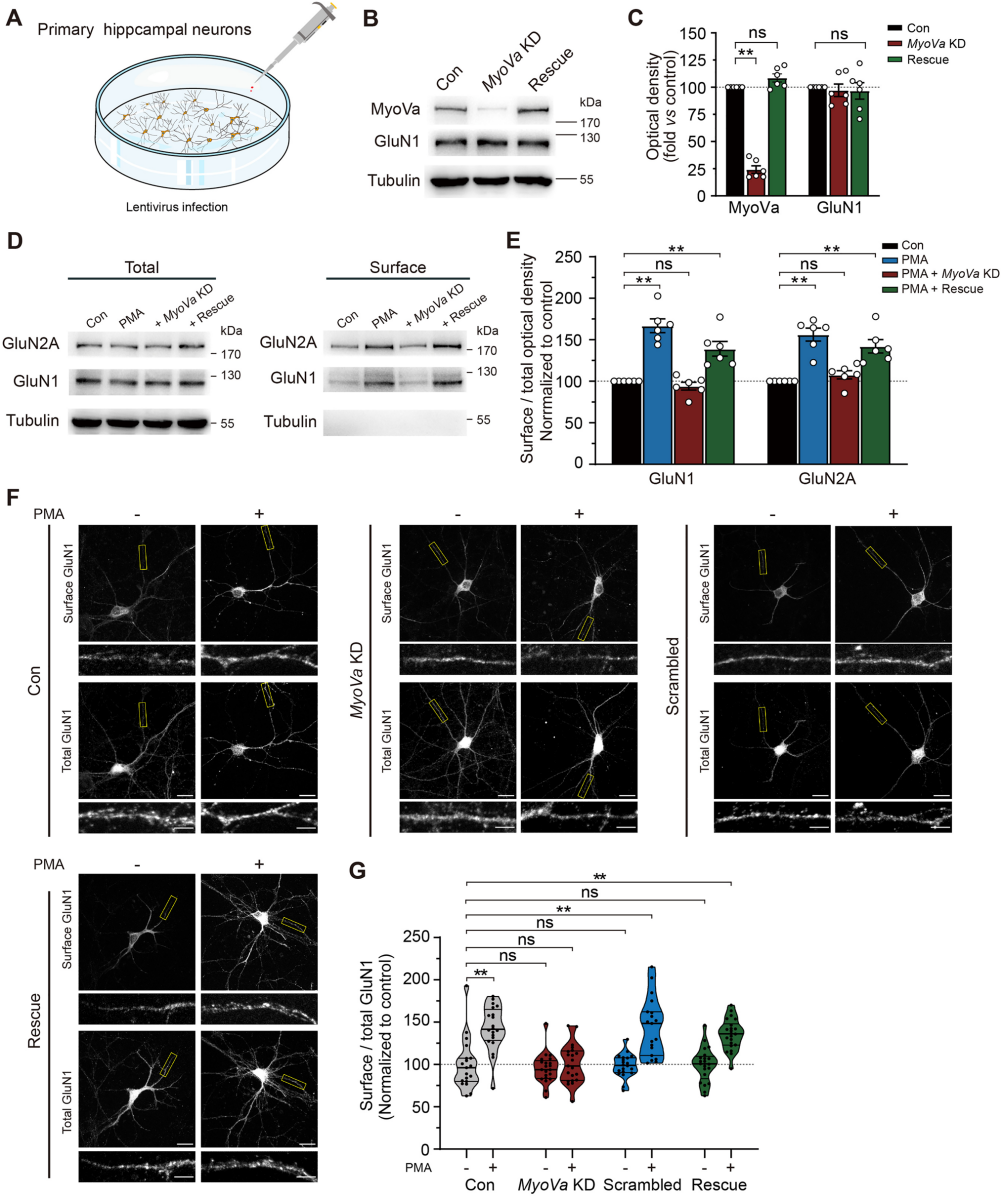
*MyoVa* KD impairs NMDAR transport in cultured hippocampal neurons. **A** Schematic showing how hippocampal primary neurons were infected with lentivirus. Transfection took place at DIV9 and results were assessed at DIV16. **B** Western blots showing interference and rescue efficiency of transfection. A marked reduction in MyoVa rather than GluN1 is evident in preparations from neurons infected with *MyoVa* shRNA. Comparable MyoVa and GluN1 expression levels occur in rescue. **C** Protein expression levels of MyoVa and GluN1 as in **B**. Data represent band intensity relative to control. MyoVa: *MyoVa* KD, 0.24 ± 0.03, *P* <0.01; rescue, 1.08 ± 0.04, *P* >0.05; GluN1: *MyoVa* KD, 0.97 ± 0.06, *P* >0.05; rescue, 0.97 ± 0.08; *P* >0.05; *n* = 6; data are from at least three independent cultures; one-way repeated-measures ANOVA *vs* control. **D** The expression of GluN1 and GluN2A on the surface is impaired in *MyoVa* KD neurons. Western blot comparing surface and total NMDAR subunit expressions under different experimental treatments. **E** Surface/total ratios of NMDAR subunits as in **D**. Data represent band intensity relative to control. GluN1: PMA, 1.67 ± 0.08, *P* <0.01; *MyoVa* KD, 0.94 ± 0.05, *P* >0.05; rescue, 1.39 ± 0.09, *P* <0.01; GluN2A: PMA, 1.56 ± 0.08, *P* <0.01; *MyoVa* KD, 1.08 ± 0.05, *P* >0.05; rescue, 1.42 ± 0.08, *P* <0.01; *n* = 6; one-way repeated-measures ANOVA *vs* control. **F** *MyoVa* KD reduces the surface expression of GluN1. Representative images of primary hippocampal neurons infected with lentivirus at DIV9. The lentivirus expressed either *MyoVa* shRNA, scrambled shRNA, or wild-type MyoVa resistant to the knockdown effect. The surface and total GluN1 subunits are immunostained with the N-terminal extracellular and the C-terminal intracellular epitope of GluN1 at DIV16. Scale bars, 20 μm and 5 μm (enlarged images). **G** Analysis of the surface to total GluN1 ratio. Each group was normalized to the value of control unstimulated neurons. Control: *n* = 19 neurons, 1.00 ± 0.07; PMA: *n* = 20 neurons, 1.43 ± 0.06, *P* <0.01; *MyoVa* KD: *n* = 21 neurons, 0.95 ± 0.04, *P* >0.05; *MyoVa* KD-PMA: *n* = 21 neurons, 1.01 ± 0.05, *P* >0.05; scrambled: *n* = 20 neurons, 0.98 ± 0.03, *P* >0.05; scrambled-PMA: *n* = 19 neurons, 1.44 ± 0.08, *P* <0.01; rescue: *n* = 21 neurons, 1.00 ± 0.04, *P* >0.05; rescue-PMA, *n* = 22 neurons, 1.35 ± 0.04, *P* <0.01; data are from at least three independent cultures; one-way repeated-measures ANOVA, *vs* control. The data are represented as the mean ± SEM, ^*^P <0.05; ^**^P <0.01. ns, no significant difference.

To investigate whether MyoVa is required for NMDAR transport, we synthesized effective shRNAs consisting of a sequence previously demonstrated to KD endogenous MyoVa (Figs 3A and S2A) [42]. The shRNAs were packed into lentivirus that infects hippocampal cells. The efficiency of *MyoVa* KD was ensured by quantitative densitometry of immunoblots (MyoVa in KD, 0.24 ± 0.03, *n* = 6; *P* <0.01; Fig. 3B, C; 0.20 ± 0.04, *P* <0.01; *n* = 4; Fig. S2B, C). By contrast, the level of MyoVa was not affected by lentivirus containing scrambled shRNAs (scrambled, 0.96 ± 0.05, *P* >0.05; *n* = 4; Fig. S2B, C). Moreover, *MyoVa* KD did not alter the overall expression of MyoVb, GluN1, and other important synaptic proteins (Fig. S2B, C).

To examine the possible role of MyoVa during the surface targeting of NMDARs, we next applied a surface biotinylation assay as well as a Western blot to primary hippocampal neurons with *MyoVa* KD. As previously reported [27, 28], PKC activation caused a rapid and substantial increase in surface rather than total GluN1 and GluN2A expression (GluN1, 1.67 ± 0.08, *P* <0.01; GluN2A, 1.56 ± 0.08, *P* <0.01; *n* = 6; Fig. 3D, E). This enhancement in surface NMDAR expression, however, was absent in *MyoVa* KD neurons (GluN1, 0.94 ± 0.05, *P* >0.05; GluN2A, 1.08 ± 0.05, *P* >0.05; *n* = 6). The enhanced levels of surface NMDARs were not affected by lentiviruses containing scrambled shRNAs which were used as controls (GluN1, 1.49 ± 0.17, *P* <0.05; GluN2A, 1.53 ± 0.12, *P* <0.01; *n* = 6; Fig. S2D, E).

We next used an immunofluorescence assay in cultured hippocampal neurons to confirm the role of MyoVa in the regulation of NMDAR surface expression [31, 35]. NMDARs expressed on the membrane surface were labeled with antibodies against the N-terminal extracellular epitope of GluN1 under membrane-impermeable conditions. The total NMDARs were labeled with antibodies against the C-terminal intracellular epitope of GluN1 under membrane-permeable conditions. The amount of surface NMDARs was determined by quantifying the intensity of GluN1 at the membrane. Consistent with previous surface biotinylation data, we detected a substantial increase in the surface level of GluN1 shortly after PMA treatment (0.5 μmol/L) (control: *n* = 19 neurons, 1.00 ± 0.07; PMA: *n* = 20 neurons, 1.43 ± 0.06, *P* <0.01; Fig. 3F, G). This enhancement in surface GluN1 was absent in *MyoVa* KD neurons (*MyoVa* KD: *n* = 21 neurons, 0.95 ± 0.04, *P* >0.05; *MyoVa* KD-PMA: *n* = 21 neurons, 1.01 ± 0.05, *P* >0.05). As a control, the enhanced level of surface GluN1 was not affected by lentivirus containing scrambled shRNAs (scrambled: *n* = 20 neurons, 0.98 ± 0.03, *P* >0.05; scrambled-PMA: *n* = 19 neurons, 1.44 ± 0.08, *P* <0.01).

To investigate whether the effect of MyoVa on the surface NMDAR level also applied to the NMDARs specifically located at postsynaptic sites, we quantified the colocalization of NMDARs with PSD-95 (postsynaptic density 95), a postsynaptic marker (Fig. S2F, G). Consistent with the above results on surface NMDAR expression, we found that PMA treatment (0.5 μmol/L) led to an enhancement of the NMDAR/PSD-95 colocalization (control: *n* = 20, 1.00 ± 0.05; PMA: *n* = 20, 1.43 ± 0.04, *P* <0.01). This enhancement, however, was absent in cultured hippocampal neurons with *MyoVa* KD (*MyoVa* KD: *n* = 18, 1.02 ± 0.05, *P* >0.05; *MyoVa* KD-PMA: *n* = 20, 0.98 ± 0.05, *P* >0.05). As a control, neurons injected with scrambled shRNAs still displayed enhanced NMDAR/PSD-95 colocalization (scrambled: *n* = 18, 1.06 ± 0.06, *P* >0.05; scrambled-PMA: *n* = 13, 1.46 ± 0.04, *P* <0.01).

To further validate the above results, we performed rescue experiments by overexpressing full-length MyoVa resistant to the knockdown effect in cultured *MyoVa* KD neurons (MyoVa in rescue, 1.08 ± 0.04, *P* >0.05; *n* = 6; Fig. 3B, C). We found that MyoVa rescue completely reversed the deficiency in PMA-induced surface GluN1 enhancement, as evaluated by both biotinylations (GluN1, 1.39 ± 0.09, *P* <0.01; GluN2A, 1.42 ± 0.08, *P* <0.01; *n* = 6; Fig. 3D, E) and immunofluorescence assays (rescue: *n* = 21 neurons, 1.00 ± 0.04, *P* >0.05; rescue-PMA, *n* = 22 neurons, 1.35 ± 0.04, *P* <0.01; Fig. 3F, G), suggesting that MyoVa is important for PMA-induced enhancement in both surface and postsynaptic expression of NMDARs in hippocampal neurons.

**Fig. 4:**
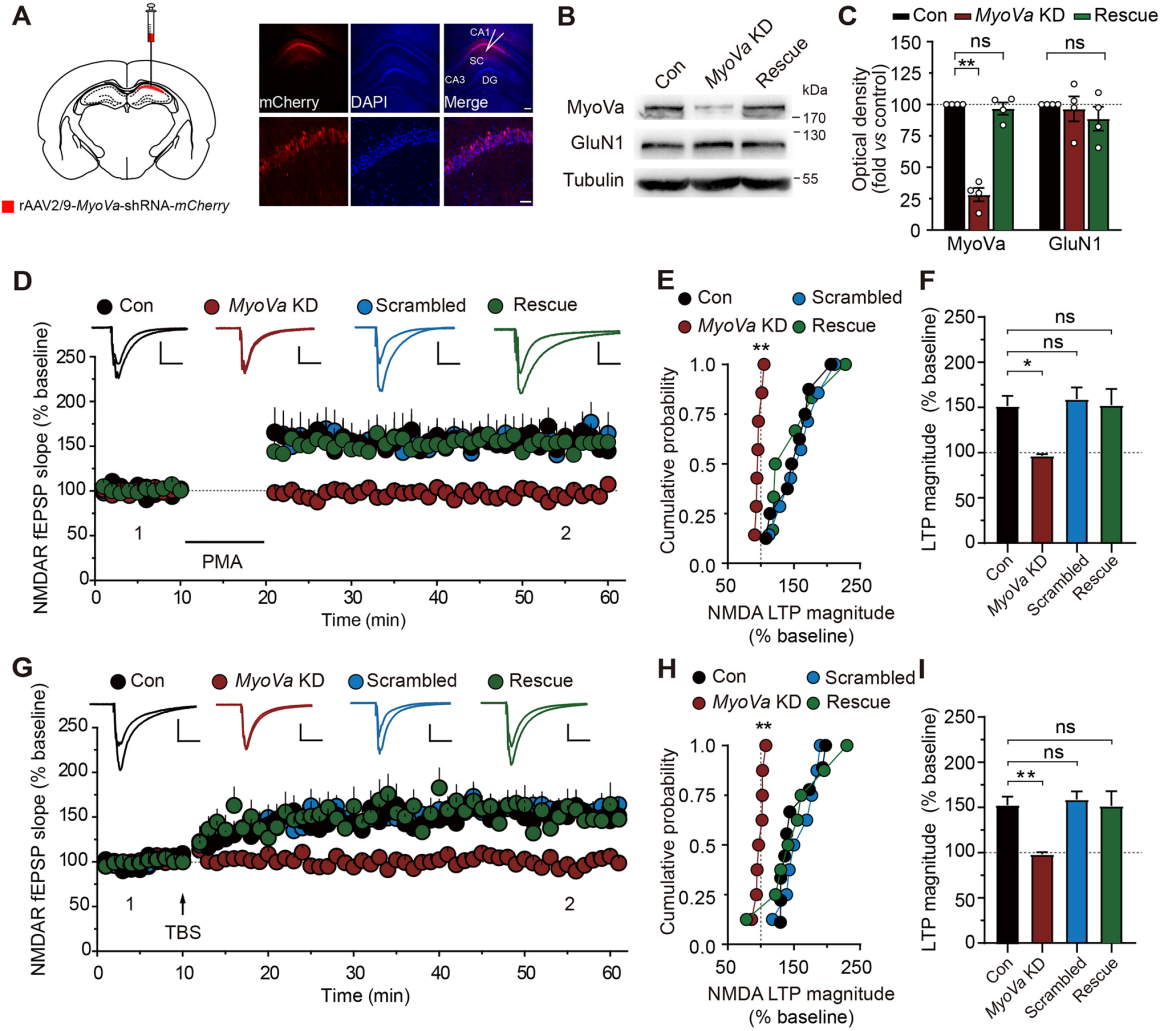
*MyoVa* KD impairs LTP of NMDA fEPSPs in acute hippocampal slices. **A** Stereotaxic injections and generation of *MyoVa* KD. Left, the locations used for unilateral viral injections at the CA1 region of the hippocampus. Right, fluorescence images of hippocampal slices expressing the red reporter (mCherry) for KD AAV expression in CA1. Scale bars, 200 μm and 50 μm (enlarged images). **B** Western blots showing the interference and rescue efficiency in the viral knockdown. A marked reduction in MyoVa rather than GluN1 occurs in preparations from neurons with *MyoVa* shRNA while MyoVa and GluN1 expression levels are comparable in rescue. **C** Protein expression levels of MyoVa and GluN1 as in **B**. Data represent band intensity relative to control. MyoVa: KD, 0.28 ± 0.05, *P* <0.01; rescue, 0.97 ± 0.04, *P* >0.05; GluN1: KD, 0.96 ± 0.10, *P* >0.05; rescue, 0.89 ± 0.10, *P* >0.05; *n* = 4; one-way repeated-measures ANOVA *vs* control. **D–F** *MyoVa* KD blocks PMA-induced LTP of NMDAR fEPSPs. The overlaid traces display changes in the average response selected at the times shown in the figure (marked by 1 and 2). **E** Cumulative probability of potentiation magnitude of NMDAR fEPSPs. **F** Summary graphs of LTP magnitude from experiments shown in **D.** Control: *n* = 8, 1.52 ± 0.11; *MyoVa* KD: *n* = 7, 0.97 ± 0.02, *P* <0.05; scrambled: *n* = 7, 1.59 ± 0.13, *P* >0.05; rescue: *n* = 6, 1.53 ± 0.18, *P* >0.05; one-way repeated-measures ANOVA *vs* control. Scale bars, 0.5 mV, 100 ms in **D**. **G–I** As in **D–F**, with the exception that LTP of NMDA fEPSPs is elicited by theta-burst stimulation (TBS). Control: *n* = 9, 1.53 ± 0.09; *MyoVa* KD: *n* = 8, 0.98 ± 0.02, *P* <0.01; scrambled: *n* = 8, 1.59 ± 0.09, *P* >0.05; rescue: *n* = 8, 1.52 ± 0.16, *P* >0.05; one-way repeated-measures ANOVA *vs* control. Scale bars, 0.5 mV, 100 ms in **G**. The data are represented as the mean ± SEM, ^*^P <0.05; ^**^P <0.01. ns, no significant difference.

The above results were obtained from cultured neurons prepared from the hippocampus of embryonic rats. These cultures, however, are likely not able to fully mimic mature synapses *in situ*. Therefore, we next determined whether MyoVa is also important for NMDAR transport in standard acute hippocampal slices when local circuits are partially maintained. One-week-old rats were stereotactically injected with AAV containing shRNAs into hippocampal CA1 (Fig. 4A) [24, 25], which suppressed the expression of MyoVa as revealed by Western blotting assays (MyoVa, 0.28 ± 0.05, *P* <0.01; *n* = 4; Fig. 4B, C). Injected rats were sacrificed 2 weeks later for fEPSP recording in the visually identified CA1 area in hippocampal slices.

Postsynaptic NMDAR transport has been reported as an important molecular mechanism underlying the LTP of NMDAR-mediated synaptic responses [9, 12, 43, 44]. We thus examined whether *MyoVa* KD could affect the LTP of NMDAR-mediated fEPSPs in hippocampal slices. In acute slices prepared from the hippocampus injected with AAV targeting MyoVa, the LTP of NMDA fEPSPs was absent, no matter whether it was induced by PMA (0.5 μmol/L; control: 1.52 ± 0.11, *n* = 8; *MyoVa* KD: 0.97 ± 0.02, *n* = 7, *P* <0.05; Fig. 4D, F) [28, 29] or TBS (control: 1.53 ± 0.09, *n* = 9; *MyoVa* KD: 0.98 ± 0.02, *n* = 8, *P* <0.01; Fig. 4G, I) [12, 26]. By contrast, the LTP of NMDA fEPSPs was intact in slices injected with scrambled shRNAs (PMA: 1.59 ± 0.13, *n* = 7, *P* >0.05; TBS: 1.59 ± 0.09, *n* = 8, *P* >0.05). Moreover, the blockade of LTP by *MyoVa* KD was reversed by the expression of shRNA-insensitive full-length MyoVa (PMA: 1.53 ± 0.18, *n* = 6, *P* >0.05; TBS: 1.52 ± 0.16, *n* = 8, *P* >0.05). In contrast, the expression of full-length MyoVb failed to restore the impaired NMDAR LTP (Fig. S3A, F), further indicating that MyoVb does not participate in NMDAR transport.

To determine whether the impaired NMDAR LTP was caused by altered basal excitatory synaptic transmission, we measured NMDAR mEPSCs and found both their amplitude and frequency were unaffected (Fig. S3G–I). Moreover, the NMDAR LTD was normal in *MyoVa* KD slices (Fig. S3J–L), which suggests that MyoVa mainly contributes to NMDAR delivery to the postsynaptic membrane rather than an LTD-related change in surface mobility. These results indicate that *MyoVa* KD blocks the LTP of NMDA fEPSPs by interrupting postsynaptic NMDAR transport.

Taken together, our results suggest that MyoVa is essential for postsynaptic NMDAR transport.

### CaMKII Regulates NMDAR Transport *via* MyoVa

MyoVa is a Ca^2+^-sensitive motor protein, as both its conformation and actin-activated ATPase activity can be regulated by micromolar Ca^2+^ [23, 45–49]. On the other hand, activation of NMDARs elicits Ca^2+^ influx and Ca^2+^ elevation in spines [50–52], which could lead to multiple downstream cascade events including the activation of various protein kinases. CaMKII is a downstream effector that is activated by Ca^2+^/CaM. Importantly, CaMKII has been reported to be associated with MyoVa [53, 54]. Interestingly, the CaMKII-MyoVa association increased during PMA treatment, which also induced NMDAR transport (Fig. S5A–D). We thus investigated whether CaMKII plays a role in regulating the MyoVa-NMDAR association and NMDAR transport.

**Fig. 5:**
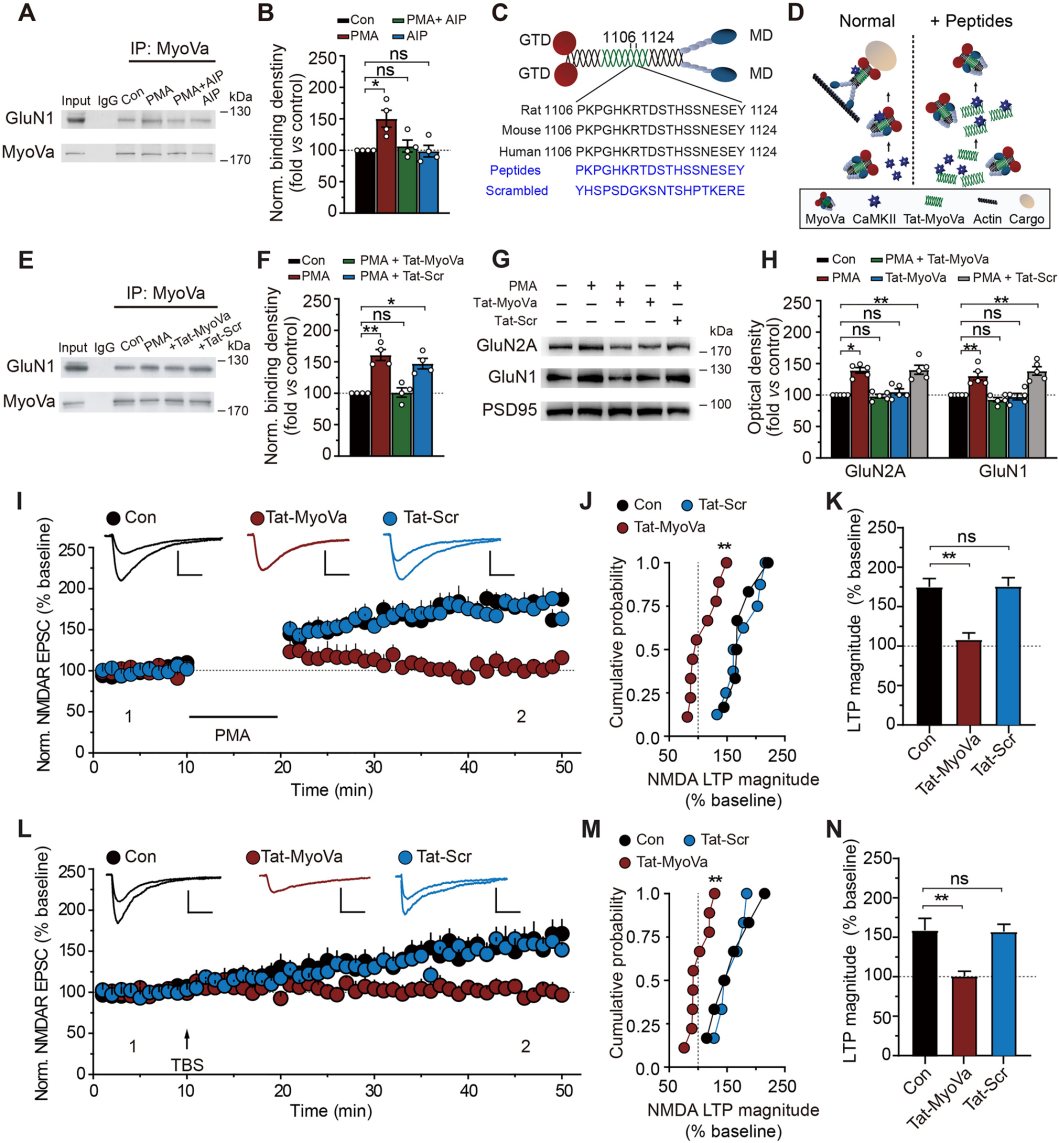
CaMKII regulates NMDAR transport *via* its interaction with MyoVa. **A** Co-IP assays with antibody against MyoVa reveals that the presence of the CaMKII-specific inhibitor AIP (1 μmol/L) reduces the MyoVa-GluN1 association. **B** Statistical analysis of the association level between GluN1 and MyoVa. The GluN1 band density was divided by the MyoVa band density and subsequently normalized to the control condition. PMA, 1.50 ± 0.13, *P* <0.05; PMA + AIP, 1.01 ± 0.09, *P* >0.05; AIP, 0.99 ± 0.01, *P* >0.05; *n* = 4; one-way repeated-measures ANOVA *vs* control. **C** Diagram of the organization and cross-species sequence alignments of MyoVa. Upper panel, the domain organization of MyoVa. The CaMKII binding domain is located in the proximal tail region of MyoVa, with some participation from adjacent regions. Lower panel, the alignments of sequences from 1106 to 1124 in the neck region of rat MyoVa, which are conserved across different species. **D** Schematic of the short cell membrane permeable peptides (Tat-MyoVa) that disrupt CaMKII-MyoVa interaction. The Tat-MyoVa is based on a CaMKII-binding sequence (1106–1124) in MyoVa. It was used to disrupt the CaMKII-MyoVa interaction by competing with MyoVa for binding to CaMKII. **E** Co-IP assays reveal that the interfering peptides Tat-MyoVa (4 μmol/L) reduce the association of MyoVa and GluN1. **F** Statistical analysis of the association level between GluN1 and MyoVa. The GluN1 band density was divided by the MyoVa band density and subsequently normalized to the control condition. PMA, 1.61 ± 0.09, *P* <0.01; PMA + Tat-MyoVa, 1.01 ± 0.07, *P* >0.05; PMA + Tat-Scr, 1.47 ± 0.08, *P* <0.05; *n* = 4; one-way repeated-measures ANOVA *vs* control. **G** Western blot showing Tat-MyoVa interfering effects on postsynaptic NMDAR expression in TIFs (Triton insoluble fractions). The interfering peptides Tat-MyoVa (4 μmol/L) suppress the PMA-induced increase in the expression of NMDARs at postsynaptic sites. By contrast, the scrambled peptides (Tat-Scr) do not affect postsynaptic NMDAR subunit expression. **H** Protein expression levels of GluN1 and GluN2A at postsynaptic sites as in **E**. NMDARs band density was normalized by dividing it by PSD95 band density, which served as a postsynaptic marker. Data represent band intensity relative to control. GluN2A: PMA, 1.30 ± 0.07, *P* <0.05; Tat-MyoVa-PMA, 0.93 ± 0.03; *P* >0.05; Tat-MyoVa, 0.97 ± 0.05; *P* >0.05; Ta-Scr-PMA, 1.38 ± 0.07, *P* <0.01; GluN1: PMA, 1.39 ± 0.05; *P* <0.01; Tat-MyoVa-PMA, 0.98 ± 0.03, *P* >0.05; Tat-MyoVa, 1.05 ± 0.05; *P* >0.05; Tat-Scr-PMA, 1.40 ± 0.07, *P* <0.01; *n* = 5; one-way repeated-measures ANOVA *vs* control. **I–K** Tat-MyoVa blocks PMA-induced LTP of NMDA EPSCs. The overlaid traces display changes in the average response selected at the times shown (marked by 1 and 2). **J** Cumulative probability of potentiation magnitude of NMDA EPSCs. **K** Summary graphs of LTP magnitude from experiments shown in **I**. Control: 1.75 ± 0.11, *n* = 6; Tat-MyoVa: 1.08 ± 0.08, *n* = 9; *P* <0.01; Tat-Scr: 1.76 ± 0.11, *n* = 8, *P* >0.05; one-way repeated-measures ANOVA *vs* control. Scale bars, 50 pA, 100 ms in **I**. **L–N** As in **I–K**, with the exception that LTP of NMDA EPSCs was induced by TBS. Control: 1.59 ± 0.15, *n* = 6; Tat-MyoVa: 1.01 ± 0.06, *n* = 9, *P* <0.01; Tat-Scr: 1.57 ± 0.09, *n* = 6, *P* >0.05; one-way repeated-measures ANOVA *vs* control. Scale bars, 50 pA, 100 ms in **L**. The data are represented as the mean ± SEM, ^*^P <0.05; ^**^P <0.01. ns, no significant difference.

Co-IP of MyoVa with NMDAR subunit GluN1 was applied to hippocampal homogenates. As expected, the MyoVa-GluN1 association was enhanced after PMA treatment (0.5 μmol/L) (Fig. 5A, B). This enhancement, however, was absent when the antagonist of CaMKII, AIP (autocamtide inhibitory peptide, 1 μmol/L), was applied (PMA, 1.50 ± 0.13, *P* <0.05; PMA + AIP, 1.01 ± 0.09, *P* >0.05; AIP, 0.99 ± 0.01, *P* >0.05; *n* = 4; Fig. 5A, B). These results suggest that CaMKII regulates the interaction of MyoVa with NMDARs during NMDAR transport.

CaMKII-dependent regulation of MyoVa-NMDAR points to CaMKII’s potential role in NMDAR transport. To test this possibility, we applied a Western blotting assay to the TIF preparation, which roughly represents postsynaptic components [31, 33, 34]. As shown in Fig. S4, PMA-induced enhancement in postsynaptic NMDAR expression was reduced by AIP treatment (Fig. S4A, B). By contrast, AIP alone failed to have any effect on postsynaptic GluN1 expression (Fig. S4A, B).

The role of CaMKII in NMDAR transport was further examined using whole-cell patch-clamp recording. Both PMA and TBS-induced LTP of NMDAR EPSCs, which have been proposed to be mediated by postsynaptic NMDAR transport, were completely abolished by perfusing acute hippocampal slices with AIP (1 μmol/L; Fig. S4C–H). Taken together with the above biochemical data, our results support the notion that CaMKII plays an important role in NMDAR transport.

Under basal conditions, CaMKII associates with MyoVa, with a consensus phosphorylation site close to this binding site [53]. Notably, this CaMKII-MyoVa association was enhanced during PMA-induced NMDAR transport, as revealed by both co-IP (Fig. S5A, D) and immunofluorescence assays (Fig. S5E, F). We speculate that CaMKII regulates NMDAR transport by increasing its association with MyoVa for subsequent switching MyoVa to an active state capable of binding cargo. To test this speculation, we design a cell-permeable short peptide based on the conservative CaMKII binding domain of MyoVa (Tat-MyoVa) that is centered around the proximal tail region (1106–1124) [53], to interfere with the CaMKII-MyoVa interaction (Fig. 5C, D). The efficiency of Tat-MyoVa in blocking the CaMKII-MyoVa interaction was confirmed by co-IP experiments on hippocampal homogenates with antibodies to MyoVa (Fig. S6A, B). Control experiments were also performed to see that Tat-MyoVa (4 μmol/L) has no influence on CaMKII activities (Fig. S6C) or NMDA EPSCs (Fig. S6D, E) under basal conditions.

Tat-MyoVa inhibition in the CaMKII-MyoVa association was accompanied by a drop in the MyoVa-GluN1 association (PMA, 1.61 ± 0.09, *P* <0.01; PMA + Tat-MyoVa, 1.01 ± 0.07, *P* >0.05; PMA + Tat-Scr, 1.47 ± 0.08, *P* <0.05; *n* = 4; Fig. 5E, F), suggesting that the CaMKII-MyoVa association affects the interaction between NMDARs and MyoVa. This supports the existence of a complex composed of CaMKII, MyoVa, and NMDARs. This CaMKII-MyoVa-NMDAR complex places CaMKII in a strategic position to activate MyoVa for cargo binding and subsequently enhance MyoVa-NMDAR interaction, which in turn facilitates PMA-induced NMDAR transport. In line with this notion, interfering with CaMKII-MyoVa association using Tat-MyoVa suppressed the PMA-induced enhancement in postsynaptic NMDAR expression (GluN2A: PMA, 1.30 ± 0.07, *P* <0.05; Tat-MyoVa-PMA, 0.93 ± 0.03; *P* >0.05; GluN1: PMA, 1.39 ± 0.05; *P* <0.01; Tat-MyoVa-PMA, 0.98 ± 0.03, *P* >0.05; *n* = 5; Fig. 5G, H), as revealed by Western blotting assays on TIF preparations that roughly represent the postsynaptic compartment [31, 33, 34]. Furthermore, both PMA- and TBS-elicited LTP of NMDA EPSCs (Fig. 5I, N) and NMDA fEPSPs (Fig. S6F, K), which have been shown to be mediated by postsynaptic NMDAR transport, were also abolished by Tat-MyoVa (4 μmol/L). As a control, peptides with scrambled sequences failed to have any significant effects on MyoVa-NMDAR associations or the LTP of NMDA EPSCs (PMA: control, 1.75 ± 0.11, *n* = 6; Tat-MyoVa, 1.08 ± 0.08, *n* = 9; *P* <0.01; Tat-Scr, 1.76 ± 0.11, *n* = 8, *P* >0.05; TBS: control, 1.59 ± 0.15, *n* = 6; Tat-MyoVa, 1.01 ± 0.06, *n* = 9, *P* <0.01; Tat-Scr, 1.57 ± 0.09, *n* = 6, *P* >0.05; Fig. 5I, N).

Together, our results suggest that CaMKII regulates NMDAR transport through its direct interaction with MyoVa.

### MyoVa Traffics NMDARs by Interacting with Rab11/FIP3

**Fig. 6:**
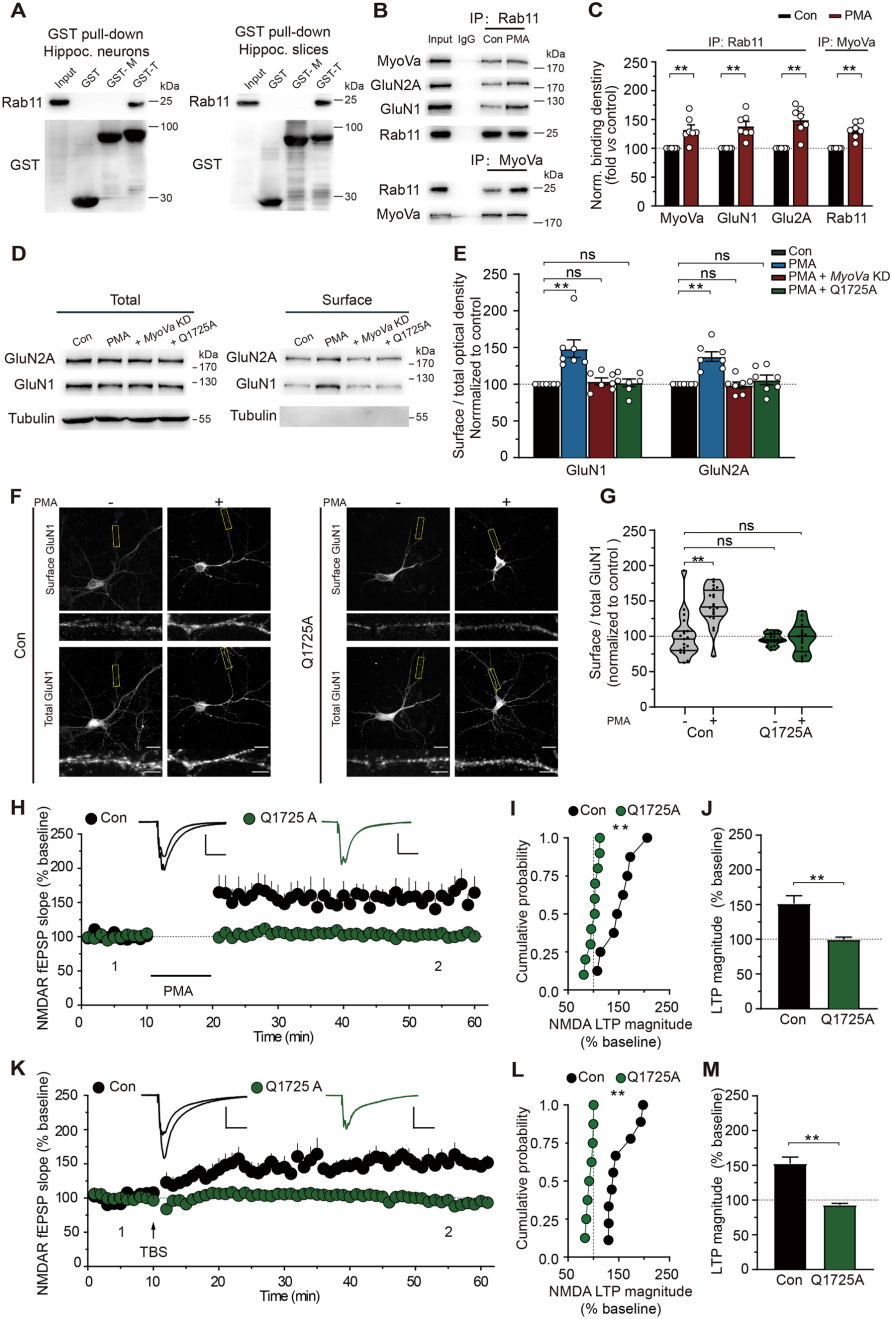
MyoVa traffics NMDARs by interacting with Rab11. **A** GST-pull-down assays. The medial tail (GST-M, M tail) or globular tail (GST-G, G tail) of MyoVa fused to GST was incubated with homogenates from hippocampal cells (left) or hippocampal slices (right). Rab11 binds with the globular tail of MyoVa (GST-G) but not its medial tail (GST-M). Plain GST was used as a control. At least three independent experiments were performed. **B** Co-IP with antibodies to Rab11 or MyoVa in control or under PMA conditions. Non-immune IgG was used as control. **C** Statistical analysis of binding from experiments as in **B**. The association between Rab11 and NMDARs or MyoVa is strengthened under PMA conditions. Signals were divided by Rab11 or MyoVa signals and normalized to control. For IP with Rab 11: *n* = 7, MyoVa, 1.33 ± 0.08, *P* <0.01; GluN1, 1.50 ± 0.09, *P* <0.01; GluN2A, 1.39 ± 0.08, *P* <0.01; for IP with MyoVa: *n* = 7, Rab11, 1.31 ± 0.05, *P* <0.01; unpaired Student’s *t*-test, *vs* control. **D** Biotin assays reveal that the expression of mutant MyoVa (Q1725A) does not reverse the deficits in NMDAR surface expression in hippocampal neurons. **E** The surface/total ratio of NMDAR subunits shown in **D.** Data reflect band intensity relative to control. GluN1: PMA, 1.49 ± 0.12, *P* <0.01; KD, 1.04 ± 0.05, *P* >0.05; Q1725A, 1.02 ± 0.05, *P* >0.05; GluN2A: PMA, 1.38 ± 0.07, *P* <0.01; KD, 0.99 ± 0.01, *P* >0.05; Q1725A, 1.06 ± 0.07, *P* >0.05; *n* = 7; one-way repeated-measures ANOVA *vs* control. **F** Representative images showing the surface and total GluN1 expression in neurons infected with lentiviral particles expressing mutant MyoVa (Q1725A) resistant to the KD effect. Scale bars, 20 μm and 5 μm (enlarged images). **G** The surface to total GluN1 ratios in control and the Q1725A mutant. Each group was normalized to the value of unstimulated control neurons. Control: *n* = 19 neurons; PMA: *n* = 20 neurons, 1.43 ± 0.06, *P* <0.01; Q1725A: *n* = 19 neurons, 0.96 ± 0.02, *P* >0.05; Q1725A-PMA: *n* = 19 neurons, 0.98 ± 0.05, *P* >0.05; data are from at least three independent cultures; one-way repeated-measures ANOVA *vs* control. The control neurons in this figure were taken from Fig. 3 for comparison. **H–J** Expressing mutant MyoVa (Q1725A) does not reverse the deficits in NMDAR-mediated LTP. The overlaid traces show changes in the average response selected at the times shown (marked by 1 and 2). **I** Cumulative probability of potentiation magnitude of NMDA fEPSPs. **J** Summary graphs of LTP magnitude from experiments shown in **H**. Control: *n* = 8, 1.52 ± 0.11; Q1725A: *n* = 10, 1.00 ± 0.03, *P* <0.01; unpaired Student’s *t*-test *vs* control. Scale bars, 0.5 mV, 100 ms in **H**. The control LTPs in this figure were taken from Fig. 4 for comparison. **K–M** As in **H–J**, with the exception that LTP of NMDA fEPSPs was elicited by TBS. Control, *n* = 9, 1.53 ± 0.09; Q1725A: *n* = 8, 0.93 ± 0.02, *P* <0.01; unpaired Student’s *t*-test *vs* control. Scale bars, 0.5 mV, 100 ms in **K**. The control LTPs in this figure were taken from Fig. 4 for comparison. The data are represented as the mean ± SEM, ^*^P <0.05; ^**^P <0.01. ns, no significant difference.

To attach to its different cargoes, the myosin uses organelle-specific receptors that often comprise Rab GTPases that are inserted in the organelle membrane by geranylgeranyl moieties [22, 55]. In particular, members of the Rab family of small GTPases have emerged as potential mediators of vesicle transport by members of the myosin V family [20, 56]. Moreover, recent studies have revealed that the small GTPase Rab11 associates with NMDARs and is required for NMDAR surface transport [57–59]. These findings raise the possibility that MyoVa traffics NMDARs by interacting with Rab11. To test this hypothesis, we first corroborated the binding of MyoVa to Rab11. We carried out GST pull-downs targeting different domains of the MyoVa C terminus and found that the MyoVa globular tail interacted with Rab11 from both cultured hippocampal neurons and hippocampal slices, whereas the medial tail of MyoVa did not (Fig. 6A).

The MyoVa-Rab11 association hints that MyoVa may use Rab11 as the adaptor protein for NMDAR transport. Thus, we next examined the possible alteration in the association of MyoVa and NMDAR with Rab11 during NMDAR transport. We used an anti-Rab11 antibody to precipitate the protein complex and to examine how the association between these molecules would change during PMA-induced NMDAR transport. We found that upon PMA treatment (0.5 μmol/L) the amounts of MyoVa and NMDAR subunits (GluN1 and GluN2A) that interacted with Rab11 (MyoVa-Rab11, GluN1-Rab11, and GluN2A-Rab11) were significantly increased (MyoVa, 1.33 ± 0.08, *P* <0.01; GluN1, 1.50 ± 0.09, *P* <0.01; GluN2A, 1.39 ± 0.08, *P* <0.01; *n* = 7; Fig. 6B, C). The finding of increased MyoVa-Rab11 association and GluN1-Rab11 association was further confirmed by standard co-IP experiments with antibodies to MyoVa (Rab11, 1.31 ± 0.05, *n* = 7, *P* <0.01; Fig. 6B, C) and GluN1 (1.53 ± 0.11, *n* = 4, *P* <0.05; Fig. S7A, B), respectively.

One recent study has characterized multiple novel Rab-MyoVa interactions and revealed that MyoVa interacts with multiple Rab GTPases of the endosomal and secretory pathways [60]. By performing a series of individual mutations on residues in MyoVa, the study further showed that the Q1750A mutant in humans selectively abolishes the MyoVa interaction with Rab11A, without any effect on the MyoVa interaction with other Rab GTPases. Therefore, we next determined whether disturbing MyoVa-Rab11 with the corresponding Q1725A mutation in rats (homologous to 1750 in humans) would affect the surface expression of NMDARs in cultured hippocampal neurons (Fig. S8A). In contrast to the dramatic restoration of surface NMDAR expression by MyoVa rescue (Fig. 3D, G), the expression of shRNA-insensitive MyoVa with the Q1725A mutation failed to reverse the jeopardized surface expression of NMDARs, determined by both surface biotinylation (GluN1, 1.02 ± 0.05; GluN2A, 1.06 ± 0.07; *n* = 7; *P* >0.05; Fig. 6D, E) and immunofluorescence assays (Q1725A, *n* = 19 neurons, 0.96 ± 0.02; Q1725A-PMA, *n* = 19 neurons, 0.98 ± 0.05; *P* >0.05; Fig. 6F, G). As a control, NMDAR surface expression without PMA stimulation was unaffected in *MyoVa* KD and Q1725A mutant neurons (Fig. S8B, C). In line with these results, in hippocampal slices with *MyoVa* KD (Fig. S8D, E) the blocked LTP of NMDA fEPSPs elicited by either PMA or TBS was not restored by MyoVa expression with the Q1725A mutation (PMA: control, 1.52 ± 0.11, *n* = 8; Q1725A, 1.00 ± 0.03, *n* = 10, *P* <0.01; TBS: control, 1.53 ± 0.09, *n* = 9; Q1725A, 0.93 ± 0.02, *n* = 8, *P* <0.01; Fig. 6H, M). Collectively, these results demonstrate that the direct interaction between MyoVa and Rab11 is crucial for NMDAR transport to the membrane.

**Fig. 7:**
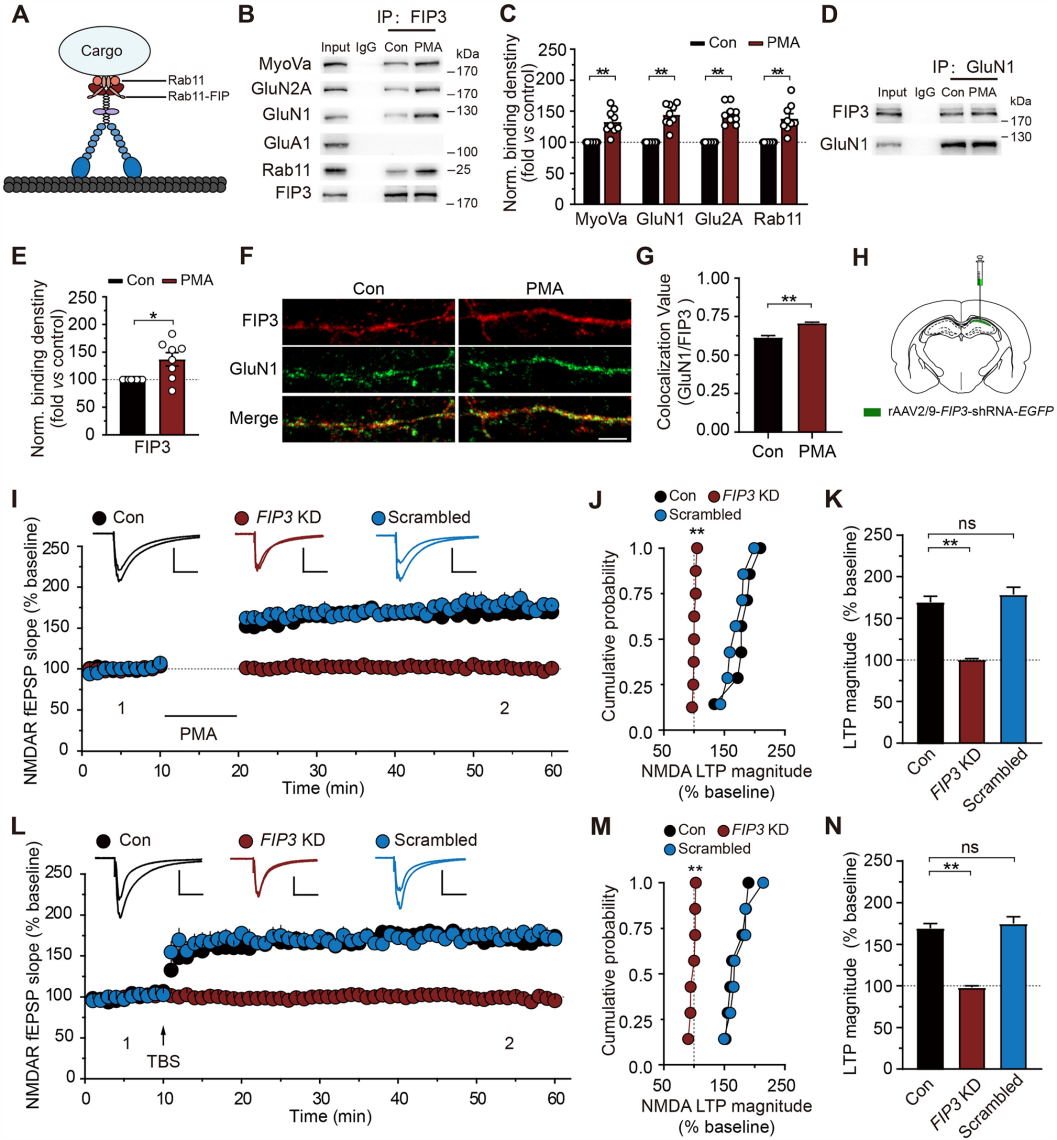
The interaction of MyoVa with FIP3 is required for NMDAR transport. **A** Schematic of MyoVa and its association with cargo proteins *via* Rab11 and FIPs. **B** Co-IP between FIP3 and associated proteins with antibodies to FIP3 in control or under PMA conditions. Non-immune IgG was used as control. **C** Statistical analysis of binding between FIP3 and associated proteins. Signals were divided by FIP3 signals and normalized to control. MyoVa: 1.33 ± 0.06, *P* <0.01; GluN1: 1.44 ± 0.06, *P* <0.01; GluN2A: 1.45 ± 0.05, *P* <0.01; Rab11: 1.38 ± 0.08, *P* <0.01; *n* = 9; unpaired Student’s *t*-test *vs* control. **D** PMA enhances the interaction of FIP3 and GluN1, revealed by co-IP between FIP3 and GluN1 with antibodies to GluN1 in control or under PMA conditions. Non-immune IgG was used as control. **E** Statistical analysis of binding between FIP3 and GluN1. FIP3 signals were divided by GluN1 signals and normalized to control. FIP3: 1.37 ± 0.12, *P* <0.05, *n* = 8; unpaired Student’s *t*-test *vs* control. **F** Representative images display increased colocalization of FIP3 and GluN1 in primary hippocampal neurons stained with GluN1 (green) and FIP3 (red) antibodies in control or under PMA conditions. Scale bar, 5 μm. **G** Quantification of the colocalization between GluN1 and FIP3 by Pearson’s coefficient. Control: *n* = 20 neurons, 0.62 ± 0.01; PMA: *n* = 26 neurons, 0.71 ± 0.01, *P* <0.01; data are from at least three independent cultures; unpaired Student’s *t*-test vs control. **H** Illustration of the locations used for unilateral viral injections into the hippocampal CA1 region. The virus expresses *FIP3* KD or scrambled shRNA and is reported by EGFP (green). **I–K** *FIP3* KD blocks the PMA-induced LTP of NMDA fEPSPs. The overlaid traces display changes in the average response selected at the times shown (marked by 1 and 2). **J** Cumulative probability of potentiation magnitude of NMDA fEPSPs.** K** Summary graphs of LTP magnitude from experiments shown in** I**. Control: *n* = 7, 1.69 ± 0.07; *FIP3* KD: *n* = 8, 1.01 ± 0.01, *P* <0.01; scrambled: *n* = 7, 1.79 ± 0.09; *P* >0.05; one-way repeated-measures ANOVA *vs* control. Scale bars, 0.5 mV, 100 ms in **I**. **L–N** As in **I–K**, with the exception that LTP of NMDA fEPSPs was elicited by TBS. Control: *n* = 7, 1.69 ± 0.06; *FIP3* KD: *n* = 7, 0.98 ± 0.02, *P* <0.01; scrambled: *n* = 7, 1.75 ± 0.08, *P* >0.05; one-way repeated-measures ANOVA *vs* control. Scale bars, 0.5 mV, 100 ms in **L**. The data are represented as the mean ± SEM, ^*^P <0.05; ^**^P <0.01. ns, no significant difference.

The ability of MyoVa to regulate NMDAR transport could thus rely on its interaction with the GTPase Rab11 and its effector Rab11-family interacting protein (Rab11-FIP, see Fig. 7A) [21, 61, 62]. Although numerous downstream effectors have been identified [55, 63], the molecular processes through which Rab11 cooperatively regulates NMDARs remain unknown. We next investigated the role of Rab11-FIPs in this process. Among the five key effectors by which Rab11 regulates endosomal trafficking, FIP3 attracted our attention, as it can interact with traffic and cytoskeleton regulators [61, 64, 65] and couples to Rab11 to mediate endosomal trafficking [66–68]. We thus investigated whether MyoVa binds to FIP3. Co-IP with anti-FIP3 antibody revealed that the interaction of FIP3 with both MyoVa and NMDARs increased during PMA-induced NMDAR transport, suggesting that MyoVa forms a complex with FIP3 to facilitate NMDAR transport (MyoVa, 1.33 ± 0.06, *P* <0.01; GluN1, 1.44 ± 0.06, *P* <0.01; GluN2A, 1.45 ± 0.05, *P* <0.01; Rab11, 1.38 ± 0.08, *P* <0.01; *n* = 9; Fig. 7B, C).

If FIP3 associates with MyoVa for NMDAR transport, then the association between FIP3 and NMDARs should also increase. To test this speculation, we carried out standard co-IP experiments with antibodies against GluN1 to assess the possible alteration in the binding of FIP3 and NMDARs (Fig. 7D, E). Our results revealed an enhanced interaction between FIP3 and NMDARs upon PMA treatment (1.37 ± 0.12, *P* <0.05, *n* = 8; Fig. 7E). In contrast, we did not detect any association between GluN1 and FIP2 (Fig. S9A).

To confirm this result, we further applied an immunofluorescence assay to cultured hippocampal neurons (Fig. 7F, G). As shown in Fig. 7F, FIP3 displayed a partial colocalization with NMDAR subunit GluN1 under basal conditions. PMA treatment (0.5 μmol/L) substantially enhanced the colocalization value (control, *n* = 20 neurons, 0.62 ± 0.01; PMA, *n* = 26 neurons, 0.71 ± 0.01, *P* <0.01; Fig. 7G), indicating that FIP3 increases its association with NMDARs during NMDAR transport. Consistent with this, *FIP3* KD decreased the co-localization of MyoVa and NMDARs, as shown by a significant reduction in the Pearson coefficient for MyoVa/GluN1 in *FIP3* KD neurons (Fig. S9B, C), further demonstrating that FIP3 is involved in NMDAR transport.

We next investigated the functional role of FIP3 in NMDAR transport by assessing the effect of *FIP3* KD on surface biotinylation assays and the LTP of NMDA fEPSPs, which has been shown to be mediated by postsynaptic NMDAR transport [7, 12, 32, 69]. As shown in Fig. S9D and E, PMA-induced enhancement of surface NMDAR expression was absent in *FIP3* KD neurons. In hippocampal slices prepared from animals injected with AAV targeting FIP3, both the LTP of NMDA fEPSPs induced by PMA (0.5 μmol/L; control: 1.69 ± 0.07, *n* = 7; *FIP3* KD: 1.01 ± 0.01, *n* = 8, *P* <0.01; scrambled: 1.79 ± 0.09, *n* = 7, *P* >0.05; Fig. 7I, K) and TBS were absent (control: 1.69 ± 0.06, *n* = 7; *FIP3* KD: 0.98 ± 0.02, *n* = 7, *P* <0.01; scrambled: 1.75 ± 0.08, *n* = 7, *P* >0.05; Fig. 7L, N). As a control, the LTP of NMDA fEPSPs was intact in slices injected with scrambled shRNAs. To assess whether the impaired NMDAR LTP is due to altered basal synaptic transmission, we measured NMDAR mEPSCs in *FIP3* KD neurons and found no changes in either mEPSC amplitude or frequency (Fig. S9F–H).

Together, our results suggest that MyoVa traffics NMDARs by interacting with Rab11/FIP3.

### *FIP3 *KD Impairs Hippocampal Memory

**Fig. 8:**
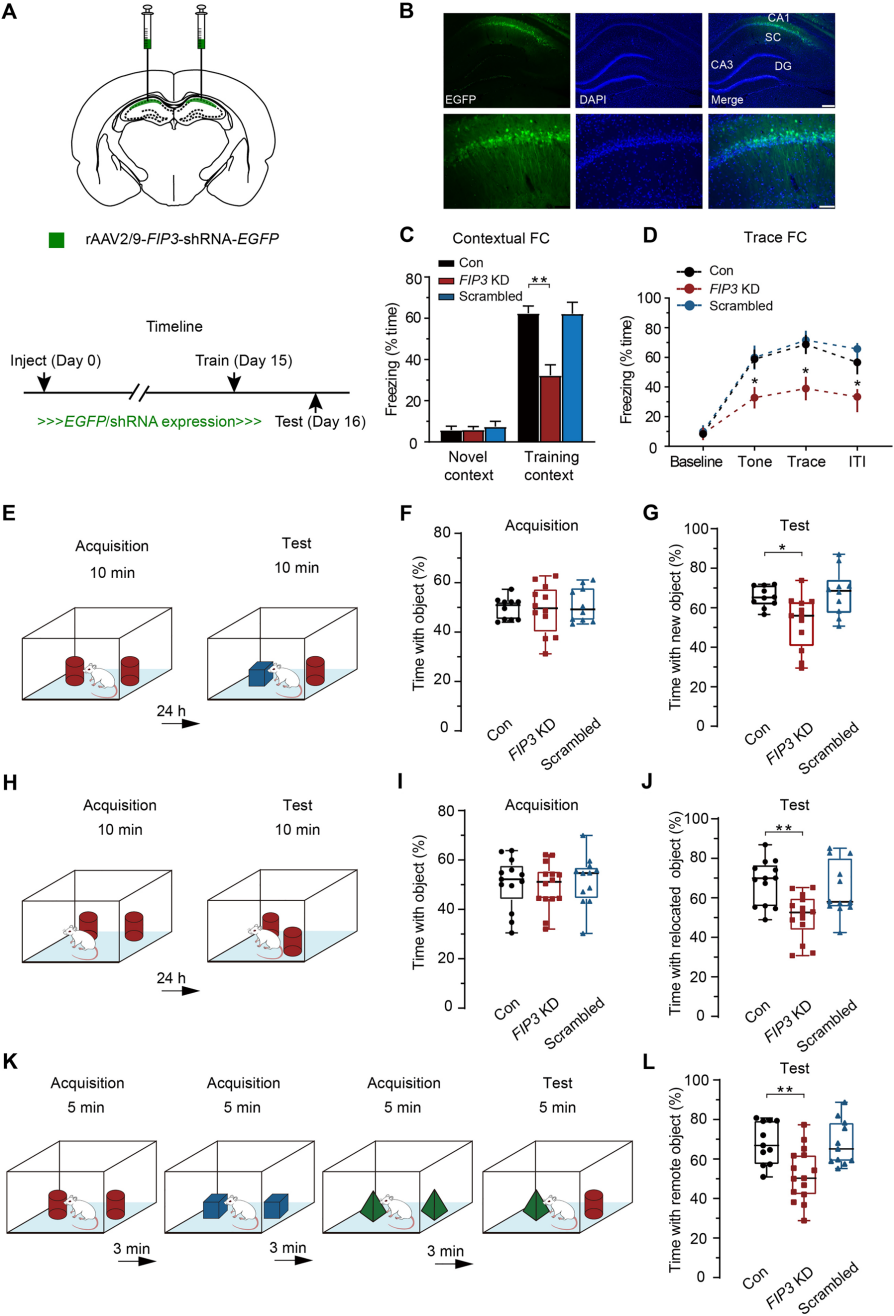
*FIP3* KD impairs hippocampal memory. **A** Schematic showing the locations used for bilateral viral injections into the CA1 region of the hippocampus. **B** Representative images showing EGFP expression in the CA1 region of the dorsal hippocampus prepared from *FIP3* KD rats. Scale bars, 250 μm and 75 μm (enlarged images). **C, D** *FIP3* KD rats show deficits in contextual (**C**) and trace fear memory (**D**) tested at 24 h, whereas the scrambled animals showed freezing comparable to control rats. FD, fear conditioning. **C**: control, *n* = 14 rats, 62.46% ± 3.52%; *FIP3* KD, *n* = 9 rats, 32.33% ± 5.13%, *P* <0.01; scrambled, *n* = 8 rats, 62.23% ± 5.56%; *P* >0.05; one-way repeated-measures ANOVA *vs* control. **D**: Control: *n* = 14 rats, tone, 58.82% ± 6.40%; trace 68.73% ± 6.40%; ITI, 56.60% ± 5.45%; *FIP3* KD: *n* = 8 rats, tone, 32.76% ± 7.21%, *P* <0.05; trace, 38.96% ± 7.94%, *P* <0.05; ITI, 33.39% ± 7.72%, *P* <0.05; scrambled: *n* = 8 rats, tone, 59.90% ± 8.03%, *P* >0.05; trace, 71.62% ± 6.31%, *P* >0.05; ITI, 65.72% ± 6.51%, *P* >0.05; two-way repeated-measures ANOVA *vs* control. **E** Schematic of the novel object preference (NOR) task. **F, G** *FIP3* KD rats show deficits in the NOR task. In the acquisition phase, the *FIP3* KD, scrambled, and control rats spend similar amounts of time exploring the two objects. In the test phase tested at 24 h, control and scrambled rats prefer the novel objects, whereas the *FIP3* KD rats exhibit a lower preference for the novel objects. Acquisition: control, *n* = 10 rats, 49.58% ± 1.43%; *FIP3* KD, *n* = 12 rats, 49.34% ± 2.87%, *P* >0.05; scrambled, *n* = 10 rats, 51.09% ± 2.21%, *P* >0.05. Test: control, *n* = 10 rats, 65.62% ± 1.67%; *FIP3* KD, *n* = 12 rats, 52.87% ± 3.92%, *P* <0.05; scrambled, *n* = 10 rats, 67.65% ± 3.70%, *P* >0.05; one-way repeated-measures ANOVA *vs* control. **H** Schematic of novel place preference test (NPP). **I, J** *FIP3* KD rats show deficits in the NPP task. In the acquisition phase of the object location task, *FIP3* KD, scrambled, and control rats spend similar time exploring the two objects. In the test phase at 24 h, control and scrambled rats prefer the relocated object, whereas the *FIP3* KD rats have a lower preference for the relocated object. Acquisition: control, *n* = 13 rats, 50.66% ± 2.88%; *FIP3* KD, *n* = 14 rats, 49.44% ± 2.48%, *P* >0.05; scrambled, *n* = 12 rats, 51.70% ± 2.90%, *P* >0.05. Test: control, *n* = 13 rats, 68.02% ± 3.12%; *FIP3* KD, *n* = 14 rats, 50.74% ± 3.01%, *P* <0.01; scrambled, *n* = 12 rats, 64.38% ± 3.92%, *P* >0.05; one-way repeated-measures ANOVA *vs* control. **K** Schematic of the temporal order memory task. **L** *FIP3* KD rats show deficits in the temporal order memory task. In the temporal order memory task, control and scrambled rats prefer the object explored at the early stage to that explored at the end, whereas the *FIP3* KD rats have a comparable preference for the two objects. Control, *n* = 11 rats, 68.31% ± 3.20%; *FIP3* KD, *n* = 15 rats, 52.14% ± 3.45%, *P* <0.01; scrambled, *n* = 11 rats, 68.07% ± 3.43%, *P* >0.05; one-way repeated-measures ANOVA *vs* control. The data are represented as the mean ± SEM, ^*^P <0.05; ^**^P <0.01.

NMDAR transport has been reported to be important for memory processes [1, 7, 12, 59, 70–72]. Taking into consideration the crucial role of MyoVa in NMDAR transport, we hypothesized that suppressing the MyoVa adaptor protein FIP3 would selectively disturb the coupling between NMDARs and MyoVa. As a result, memory formation could be affected. To test this hypothesis, we stereotactically injected AAV containing shRNAs targeting FIP3 in the dorsal hippocampal CA1 region (Fig. 8A) of 8-week-old rats. The efficiency of *FIP3* KD was then validated (KD: FIP3, 0.33 ± 0.05, *n* = 5, *P* <0.01; Figs 8B and S9I, J). The animals were allowed to recover for two weeks after the AAV injection, and then were trained and tested on the following days. *FIP3* KD rats displayed normal locomotion and motivation as indicated by the absence of any differences in distance traveled, mean speed, and mobile time in open-field analysis (Fig. S9K–N). We then conducted various memory tasks that are classically associated with hippocampal functions to evaluate the memory processes of *FIP3* KD rats [13, 36–38, 59, 73, 74].

We first assessed associative memory by contextual and trace auditory fear conditioning tasks (Fig. S9O, P). Upon re-exposure to the training context 24 h later after conditioning, the conditioned rats with *FIP3* KD displayed significantly less context-evoked freezing behavior (control, *n* = 14 rats, 62.46% ± 3.52%; *FIP3* KD, *n* = 9 rats, 32.33% ± 5.13%, *P* <0.01; scrambled, *n* = 8 rats, 62.23% ± 5.56%; *P* >0.05; Fig. 8C). Moreover, deficits in conditioned freezing behavior during the trace interval and the inter-trial intervals were also detected in the trace fear conditioning task (control: *n* = 14 rats, tone, 58.82% ± 6.40%; trace 68.73% ± 6.40%; ITI, 56.60% ± 5.45%; *FIP3* KD: *n* = 8 rats, tone, 32.76% ± 7.21%; trace, 38.96% ± 7.94%; ITI, 33.39% ± 7.72%; *P* <0.05; scrambled: *n* = 8 rats, tone, 59.90% ± 8.03%; trace, 71.62% ± 6.31%; ITI, 65.72% ± 6.51%; *P* >0.05; Fig. 8D), suggesting that FIP3 deficiency caused the deficits in fear memory processes.

Next, we assessed the recognition memory of *FIP3* KD rats using the NOR task (Fig. 8E). It has been shown that the dorsal hippocampus plays an important role during NOR memory formation, especially when spatial or contextual information is relevant [75, 76]. We found that both control and *FIP3* KD rats spent a similar amount of time exploring two identical objects during training sessions (control, *n* = 10 rats, 49.58% ± 1.43%; *FIP3* KD, *n* = 12 rats, 49.34% ± 2.87%; scrambled, *n* = 10 rats, 51.09% ± 2.21%; *P* >0.05; Fig. 8F), indicating normal locomotor abilities and levels of curiosity in *FIP3* KD rats to explore the objects. In retention tests, one of the objects used in the training session was replaced with a novel object, and animals were allowed to explore the vicinity for 10 min. In 1-day retention sessions, both control and scrambled KD rats displayed a significant preference for the novel object, whereas *FIP3* KD rats exhibited lower preference for the novel object (control, *n* = 10 rats, 65.62% ± 1.67%; *FIP3* KD, *n* = 12 rats, 52.87% ± 3.92%, *P* <0.05; scrambled, *n* = 10 rats, 67.65% ± 3.70%, *P* >0.05; Fig. 8G).

We also applied the NPP test, which is similar to the NOR task and is known to rely on the hippocampus, to measure the recognition memory of *FIP3* KD rats (Fig. 8H). In the encoding phase, *FIP3* KD rats showed performance comparable to groups of control/scrambled rats and spent similar amounts of time exploring the two identical objects (control, *n* = 13 rats, 50.66% ± 2.88%; *FIP3* KD, *n* = 14 rats, 49.44% ± 2.48%; scrambled, *n* = 12 rats, 51.70% ± 2.90%; *P* >0.05; Fig. 8I). One of the objects was then moved to a novel place and rats were returned 24 h later for another round of exploration, during which the time spent investigating each object was measured. In the test phase, the *FIP3* KD rats failed to show a preference for the moved object (*FIP3* KD, *n* = 14 rats, 50.74% ± 3.01%, *P* <0.01; Fig. 8J). By contrast, control and scrambled rats showed a significant preference to explore the moved object (control, *n* = 13 rats, 68.02% ± 3.12%; scrambled, *n* = 12 rats, 64.38% ± 3.92%, *P* >0.05).

Finally, we assessed a temporal order memory task in *FIP3* KD rats (Fig. 8K), a task that has been shown to depend on hippocampal CA1 function [77]. In the test phase, control and scrambled rats preferred the object explored earlier to that explored later (control, *n* = 11 rats, 68.31% ± 3.20%; scrambled, *n* = 11 rats, 68.07% ± 3.43%, *P* >0.05), whereas the *FIP3* KD rats showed a comparable preference, indicating impaired performance of *FIP3* KD rats in this task (*FIP3* KD, *n* = 15 rats, 52.14% ± 3.45%, *P* <0.01; Fig. 8L).

Altogether, these results suggest that FIP3 deficiency in the CA1 region, which likely impedes NMDAR transport, causes deficits in hippocampus-associated memories.

## Discussion

### MyoVa and Its Interaction with NMDAR is Essential for NMDAR Transport

Dendritic transport of NMDARs depends on motor proteins that belong to the kinesin superfamily (KIFs). Specifically, KIF3B and KIF17 are respectively required for the transport of GluN2A and GluN2B subunits [10, 13, 18, 78–80]. Before entering the dendritic spine, NMDARs need to be released from KIF and then transferred to myosin proteins that are associated with F-actin. Corresponding to the “track switch” from microtubule to actin, a specific type of myosin protein is required for NMDAR transport in the dendritic spine. Studies of three subtypes of class V myosins (Va, Vb, and Vc) have revealed dominant expression of MyoVa and MyoVb in neuronal tissue [81, 82]. The structures of MyoVa and MyoVb in mammals exhibit substantial similarity [20, 22]. They both include a head motor domain to bind actin filaments, hydrolyzing ATP and generating force, a neck domain with six calmodulin-binding IQ motifs, and a tail domain with a proximal coiled-coil sequence and a distal globular region. We here demonstrate that activity-dependent NMDARs in response to external stimuli (PKC activation by PMA or electrical inputs by TBS), which underlies the LTP of NMDAR-mediated synaptic responses [6, 28, 44], require MyoVa and MyoVa-associated adaptor proteins. This does not contradict the absence of change in spontaneous or miniature NMDAR-mediated currents [83] or in NMDAR surface expression [84], as all these results were obtained in Flailer mutant mice under baseline conditions.

By applying biochemical or electrophysiological assays to primary cultured hippocampal neurons and acute hippocampal slices, in combination with genetic manipulation techniques and behavioral tests, we demonstrated that MyoVa is the myosin protein that implements NMDAR transport to the postsynaptic membrane (for complete schematics see Fig. S10). Given that MyoVa and MyoVb are structurally similar, we also examined the possible role of MyoVb in NMDAR transport. The absence of MyoVb association with NMDARs under both basal conditions and PMA-induced NMDAR transport does not support this possibility. This could be due to the differences in protein sequences between MyoVa and MyoVb. Overall, MyoVb exhibits 62% homology with MyoVa in the full length and 74% in the motor head domain [85]. The sequence homology between MyoVb and MyoVa is in line with their similar properties. However, the distinct sequences also have a significant impact on their respective properties. For example, the sequence of loop 2 in the motor domain of MyoVb differs considerably from that of MyoVa, and this variation has been suggested to affect the actin-binding properties of myosin [85]. Therefore, these distinctions between MyoVa and MyoVb may affect their ability to transport specific types of cargo. In this scenario, MyoVa may possess a specific sequence or structure necessary for NMDAR transport, while MyoVb may lack this particular feature.

As for the MyoVa transport of NMDAR, we provide a set of evidence to support this conclusion. Firstly, under basal conditions, MyoVa interacts with NMDARs through its cargo binding domain. Their association increased during NMDAR transport. Secondly, *MyoVa* KD suppressed PMA-induced NMDAR transport. Accordingly, the LTP of NMDAR fEPSPs, which is reported to be mediated by postsynaptic NMDAR transport, is absent in *MyoVa* KD rats. It is worth mentioning that *MyoVa* KD did not affect NMDAR LTD. MyoVa moves from the minus end to the plus end of actin filaments in dendritic spines. This unidirectional movement limits MyoVa to mainly mediating transport processes towards the cell surface, such as exocytosis. In contrast, NMDAR LTD at CA1 synapses requires the receptor to move away from the synaptic site, which is dependent on actin depolymerization and the lateral diffusion of NMDARs. This difference could at least partially explain the finding that *MyoVa* KD did not influence NMDAR LTD in the hippocampal CA1 region. Thirdly, consistent with the fact that MyoVa is a Ca^2+^-sensitive motor protein, we revealed that CaMKII, the protein kinase that can be activated by increased Ca^2+^/CaM upon NMDAR activation, regulates NMDAR transport through its direct action on MyoVa, which in turn facilitates the interaction between MyoVa and NMDAR for NMDAR transport. Fourthly, we demonstrated that MyoVa traffics NMDARs by interacting with Rab11/FIP3. Rab11/FIP3 functions as the adaptor protein to couple NMDARs and MyoVa during their transport. We report that *FIP3* KD impairs various memory processes that depend on hippocampal function. Together, these findings indicate that both MyoVa and its association with NMDARs are essential for NMDAR transport.

### CaMKII-Dependent Regulation of NMDAR Transport

We reveal in the present study that CaMKII is the key player that regulates NMDAR transport. CaMKII is an effector kinase central to synaptic plasticity, learning, and memory [70, 86–90]. During synaptic and behavioral plasticity, the activation of CaMKII by increased Ca^2+^ influx facilitates the interaction among MyoVa, NMDAR, and CaMKII. Each of the three molecules interacts with the other two and thus tends to form a complex. MyoVa establishes tighter associations with NMDARs for NMDAR transport. This interaction between MyoVa and NMDARs is closely regulated by CaMKII *via* its direct action on MyoVa, as the increased MyoVa-NMDAR association can be reversed by blocking CaMKII activity with AIP. Furthermore, interfering with the CaMKII-MyoVa interaction with a short peptide (Tat-MyoVa) suppressed PMA-induced enhancement in postsynaptic NMDAR expression, as well as the LTP of NMDA EPSCs mediated by postsynaptic NMDAR transport. These findings are consistent with the widely accepted notion that MyoVa is the Ca^2+^-sensitive motor protein. For example, Ca^2+^ at micromolar concentrations can cause the unfolding of MyoVa [46, 48, 49, 54, 91]. We speculate that the Ca^2+^/CaM-activated CaMKII forms a tight association with MyoVa to facilitate its action on the neighboring phosphorylation site. This phosphorylation in turn triggers a conformational switch of MyoVa that exposes the globular tail domain (GTD) and forms a tight MyoVa-NMDAR association for NMDAR transport. Alternatively, CaMKII could directly phosphorylate GluN2A for NMDAR transport, as suggested by a recent study [92].

### Role of Rab11 and FIP3 in NMDAR Transport and Memory Formation

In the present study, we also identified the GTPase Rab11 as the adaptor protein that couples myosin Va with its NMDAR cargo. The ability of MyoVa to regulate NMDAR transport depends on the interaction of its GTD with Rab11 and its effector FIP3. Several lines of evidence support this conclusion. Firstly, whereas MyoVa rescued the effect of *MyoVa* KD, the expression of MyoVa with the Q1725A mutation that selectively abolished the MyoVa interaction with Rab11 did not. These findings support a requirement for the association of MyoVa with Rab11 for NMDAR transport. Secondly, using a combination of biochemical, immunofluorescent, and electrophysiological assays, we demonstrated that both Rab11 and FIP3 associate with MyoVa and are required for PMA- or TBS-induced transport of NMDARs to the plasma membrane. Third, we report that *FIP3* KD impairs various types of memory processes that depend on hippocampal function. Specifically, the four types of hippocampal memory tasks, i.e., fear memory, NOR, NPP, and temporal order memory, were measured. Considering the essential role of NMDAR transport in memory consolidation, the memory deficit in *FIP3* KD animals is consistent with the involvement of FIP3 in MyoVa-dependent NMDAR transport. We thus propose that Rab11 and FIP3 function as key adaptor proteins to couple MyoVa and NMDAR for NMDAR transport (Fig. S10).

### Supplementary Information

Below is the link to the electronic supplementary material.

Supplementary file1 (PDF 1884 KB)
