# Supplementary material for: Myosin Va-dependent Transport of NMDA Receptors in Hippocampal Neurons
Source: Neurosci Bull. 2024 Jan 30;40(8):1053–75. doi: 10.1007/s12264-023-01174-y (PMC11306496; doi:10.1007/s12264-023-01174-y)
Supplement: Supplementary file 1 — Supplementary file1 (PDF 1884 KB) [file 12264_2023_1174_MOESM1_ESM.pdf]

Supplementary Materials

Supplementary Figures

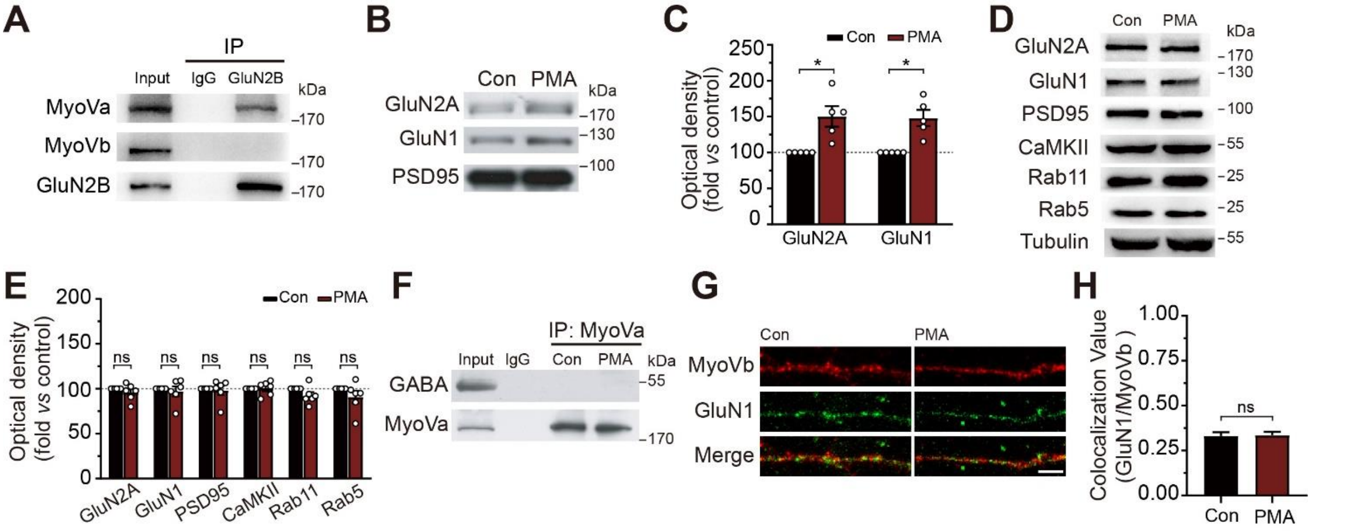

Fig. S1 PMA (phorbol myristate acetate) induces enhancement in NMDAR expression at postsynaptic sites. **A** Co-IP experiment with antibodies against GluN2B reveals an association with MyoVa, but not with MyoVb. Non-immune IgG was used as a control. The experiment was performed independently at least 3 times. **B** Western blots showing enhanced NMDAR expression in the TIF (Triton insoluble fraction) of a hippocampal slice under PMA treatment. **C** The protein expression levels of GluN2A and GluN1 in **A**. NMDAR band density was normalized by dividing it by PSD95 band density, which served as a postsynaptic marker. Data represent band intensity relative to control. GluN2A: 1.50 ± 0.14, *P* <0.05; GluN1: 1.48 ± 0.11, *P* <0.05; *n* = 5; unpaired Student's *t*-test *vs* control. **D** Western blots showing that PMA treatment does not change the total amount of protein expression in hippocampal neurons. **E** The protein expression levels in **C**. Data represent band intensity relative to control. GluN2A: 0.96 ± 0.04, *P* >0.05; GluN1: 0.97 ± 0.06, *P* >0.05; PSD95: 0.98 ± 0.05, *P* >0.05; CaMKII: 1.01 ± 0.03, *P* >0.05; Rab11: 0.92 ± 0.04, *P* >0.05; Rab5: 0.91 ± 0.07, *P* >0.05; *n* = 6; unpaired Student's *t*-test *vs* control. **F** Co-IP experiments with antibodies against MyoVa reveal that GABARs do not bind to MyoVa. Non-immune IgG was used as a control. The experiment was performed independently at least 3 times. **G** Representative images of primary hippocampal neurons stained with

MyoVb (red) and GluN1 (green) antibodies in control or under PMA conditions. Scale bar, 5 μm. **H** Quantification of the colocalization between GluN1 and MyoVb by Pearson's coefficient. Control: n = 26 neurons, 0.33 ± 0.02; PMA: n = 29 neurons, 0.34 ± 0.02, P >0.05, data are from at least three independent cultures; unpaired Student's t-test *vs* control. The data are represented as the mean ± SEM, $^{*}P$ <0.05; ns, no significant difference.

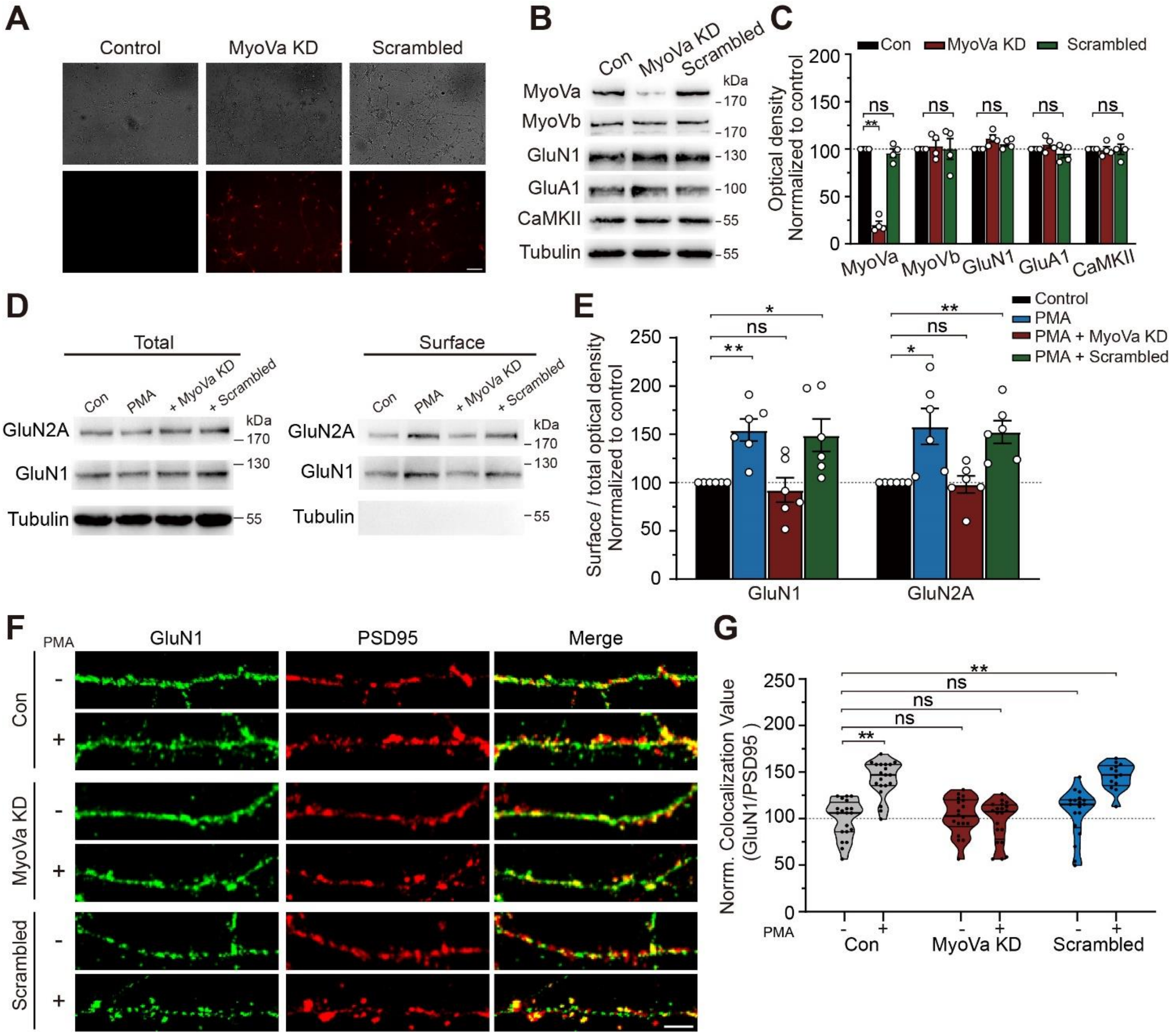

Fig. S2 MyoVa KD impairs PMA-induced membrane delivery of NMDARs. **A** Fluorescence images showing the expression of mCherry in KD (middle column) and scrambled lentivirus-infected cells (right column). The upper row shows bright field images while the lower row shows mCherry fluorescence images. Scale bar, 200 μm. **B** Western blot results indicating that the scrambled lentivirus

does not affect the expression of MyoVa. Comparatively, the level of MyoVa is substantially reduced by lentivirus-containing shRNAs targeting endogenous MyoVa. Moreover, both MyoVa KD and scrambled do not alter the overall expression of MyoVb, GluN1, GluA1, and CaMKII. **C** Statistical quantification of the results in **B**. Data represent band intensity relative to control. MyoVa: MyoVa KD, 0.20 ± 0.04, P <0.01; scrambled, 0.96 ± 0.05, P >0.05; MyoVb: MyoVa KD, 1.03 ± 0.06, P >0.05; scrambled, 1.00 ± 0.11, P >0.05; GluN1: MyoVa KD, 1.11 ± 0.16, P >0.05; scrambled, 1.06 ± 0.30; P >0.05; GluA1: MyoVa KD, 1.05 ± 0.04, P >0.05; scrambled, 0.95 ± 0.04; P >0.05; CaMKII: MyoVa KD, 0.99 ± 0.04, P >0.05; scrambled, 1.00 ± 0.05; P >0.05; n = 4; one-way repeated-measures ANOVA *vs* control. **D** The scrambled lentivirus has no effect on the surface expression of GluN1 and GluN2A, revealed by surface biotinylation assays in primary hippocampal neurons. In contrast, MyoVa KD decreases NMDAR surface expression. **E** The surface/total ratio of NMDAR subunits shown in **D**. Data represent band intensity relative to control. GluN1: PMA, 1.55 ± 0.11, P <0.01; MyoVa KD, 0.92 ± 0.13, P >0.05; scrambled, 1.49 ± 0.17, P <0.05; GluN2A: PMA, 1.58 ± 0.19, P <0.05; MyoVa KD, 0.98 ± 0.09, P >0.05; scrambled, 1.53 ± 0.12, P <0.01; n = 6; one-way repeated-measures ANOVA *vs* control. **F** PMA-induced enhancement in PSD95 (red)/GluN1 (green) colocalization is absent in cultured hippocampal neurons with MyoVa KD. Scale bar, 5 μm. **G** Plots showing normalized colorization values in **F.** PMA treatment significantly enhances GluN1 colocalization with PSD95 in control and scrambled but not in MyoVa KD cultures as demonstrated by Pearson's coefficient normalized to control. Control: n = 20, 1.00 ± 0.05; PMA: n = 20, 1.43 ± 0.04, P <0.01; MyoVa KD: n = 18, 1.02 ± 0.05, P >0.05; MyoVa KD-PMA: n = 20, 0.98 ± 0.05, P >0.05; scrambled: n = 18, 1.06 ± 0.06, P >0.05; scrambled-PMA: n = 13, 1.46 ± 0.04, P <0.01; from at least three independent cultures. One-way repeated-measures ANOVA *vs* control. The data are represented as the mean ± SEM, $^{*}P$ <0.05; $^{**}P$ <0.01; ns, no significant difference.

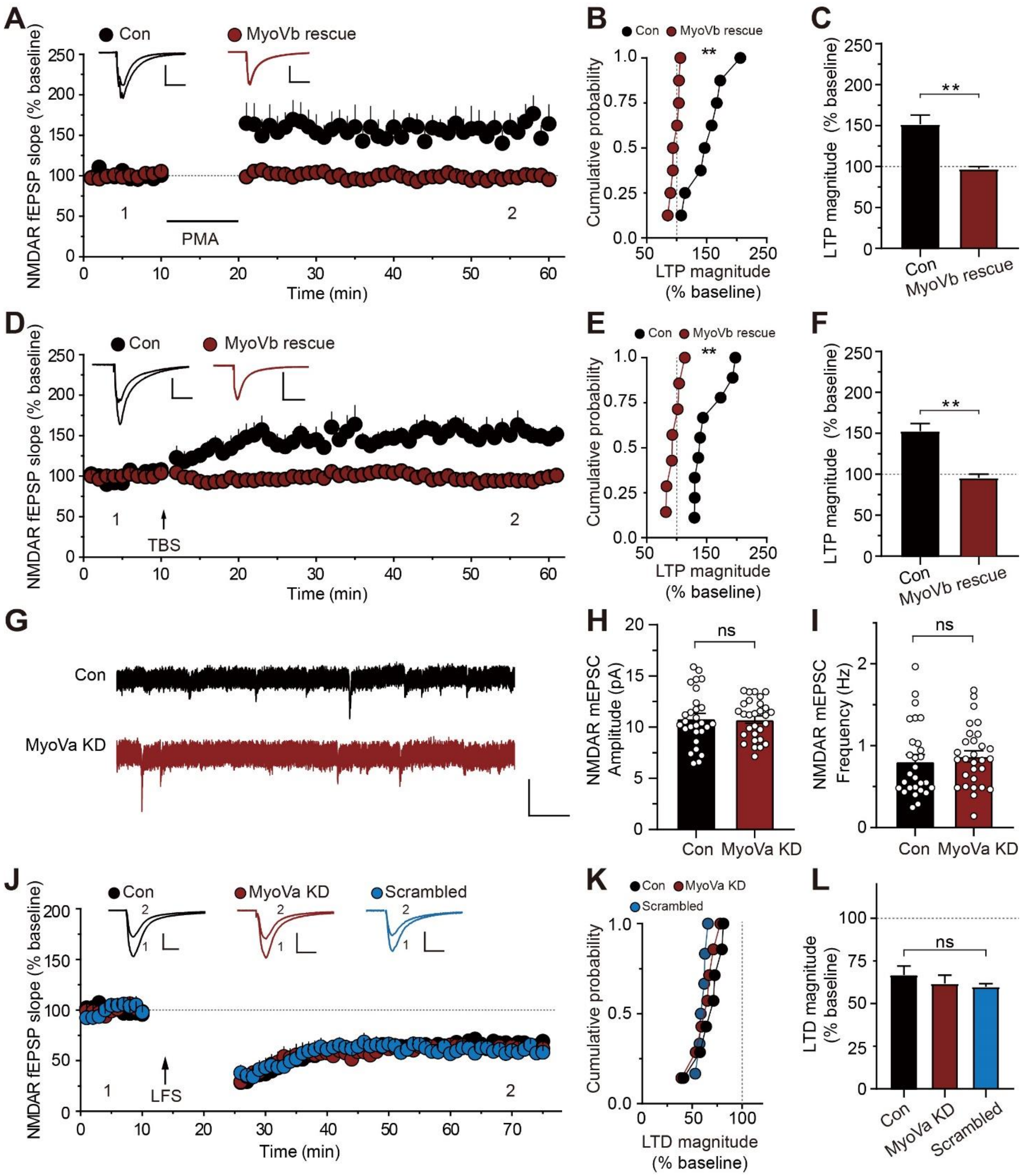

Fig. S3 The effect of MyoVa KD on mEPSC and NMDAR-dependent LTD. **A–C** Expressing MyoVb does not reverse the deficits in NMDAR-mediated LTP. The overlaid traces show changes in the average response selected at the times shown (marked by 1 and 2). **B** Cumulative probability of potentiation magnitude of NMDA fEPSPs. **C** Summary graphs of LTP magnitude from experiments shown in **A**. Control: $n = 8$, 1.52 ± 0.11; MyoVb: $n = 8$, 0.97 ± 0.03, P <0.01; unpaired Student's t-test

vs control. Scale bars, 0.5 mV, 100 ms in **A**. The control LTPs in this figure were taken from Fig. 4 for comparison. **D–F** As in **A–C**, with the exception that LTP of NMDA fEPSPs was elicited by TBS. Control, $n = 9$, 1.53 ± 0.09; MyoVb: $n = 7$, 0.96 ± 0.04, $P < 0.01$; unpaired Student's *t*-test *vs* control. Scale bars, 0.5 mV, 100 ms in **D**. The control LTPs in this figure were taken from Fig. 4 for comparison. **G–I** MyoVa KD does not affect the amplitude and frequency of NMDAR mEPSCs. Representative mEPSC traces are shown in **G**; mEPSC amplitude and frequency are shown in **H** and **I**, respectively. Amplitude (**H**): control: $n = 28$, 10.84 ± 0.70; MyoVa KD: $n = 29$, 10.75 ± 0.37, $P > 0.05$. Frequency (**I**): control: $n = 28$, 0.81 ± 0.08; MyoVa KD: $n = 29$, 0.87 ± 0.07, $P > 0.05$; unpaired Student's *t*-test *vs* control. Scale bars, 10 pA, 2s in **G**. **J–L** MyoVa KD does not affect LTD of NMDA fEPSPs. The overlaid traces display changes in the average response selected at the times shown (marked by 1 and 2). **K** Cumulative probability of depression magnitude of NMDA fEPSPs. **L** Summary graphs of LTD magnitude from experiments shown in **J**. Control: $n = 7$, 0.67 ± 0.05; MyoVa KD: $n = 7$, 0.62 ± 0.05, $P > 0.05$; scrambled: $n = 6$, 0.60 ± 0.02, $P > 0.05$; one-way repeated-measures ANOVA *vs* control. Scale bars, 0.5 mV, 50 ms in **J**. The data are represented as the mean ± SEM, $^{**}P < 0.01$; ns, no significant difference.

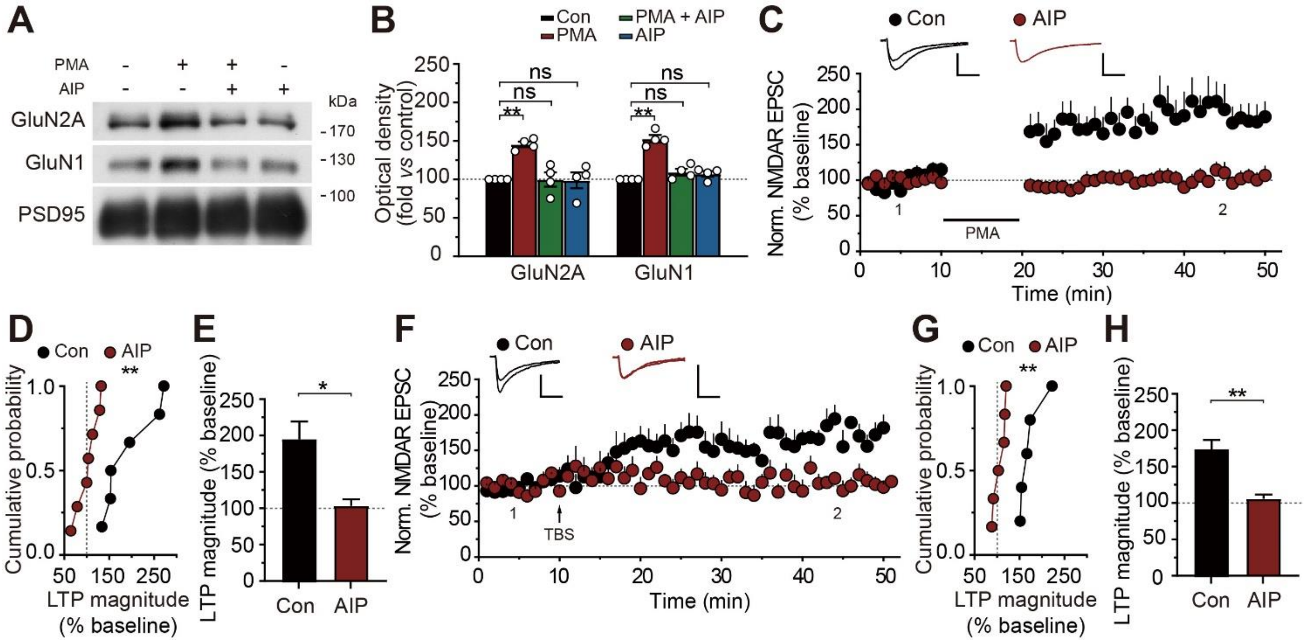

Fig. S4 Inhibition of CaMKII suppresses NMDAR surface expression and NMDAR LTP. **A** Western blots showing that the PMA-induced increase in postsynaptic NMDAR expression is prevented by the CaMKII-specific inhibitor AIP (1 μmol/L). Bands demonstrate expression levels of GluN1 and GluN2A in TIF. **B** The protein expression levels of GluN2A and GluN1 in **A**. AIP successfully reverts the PMA-induced NMDAR expression rise back to control levels. Data represent band intensity relative to control. GluN2A: PMA, 1.46 ± 0.04, P <0.01; AIP-PMA, 1.00 ± 0.09, P >0.05; AIP, 0.99 ± 0.10, P >0.05; GluN1: PMA, 1.53 ± 0.05, P <0.01; AIP-PMA, 1.09 ± 0.04, P >0.05; AIP, 1.07 ± 0.07, P >0.05; n = 4; one-way repeated-measures ANOVA *vs* control. **C–E** AIP (1 μmol/L) blocks PMA-induced potentiation in NMDAR EPSCs. Peak amplitudes of EPSCs are normalized to baseline and presented as percentage ratios. The overlaid traces display changes in the average response selected at the times shown (marked by 1 and 2). **D** Cumulative probability of potentiation magnitude of NMDA EPSCs. **E** Summary graphs of LTP magnitude from experiments shown in **C**. Control, 1.95 ± 0.24, n = 6; AIP, 1.03 ± 0.09, P <0.05, n = 7; unpaired Student's t-test *vs* control. Scale bars, 50 pA, 100 ms in **C**. **F–H** As in **C–E** with the exception that LTP of NMDAR EPSCs was elicited by TBS. Control, 1.74 ± 0.13, n = 5; AIP, 1.06 ± 0.06, P <0.01, n = 6; unpaired Student's t-test *vs* control. Scale bars, 50 pA and 100 ms in **F**. The data are represented as the mean ± SEM, $^{*}P$ <0.05; $^{**}P$ <0.01; ns, no significant difference.

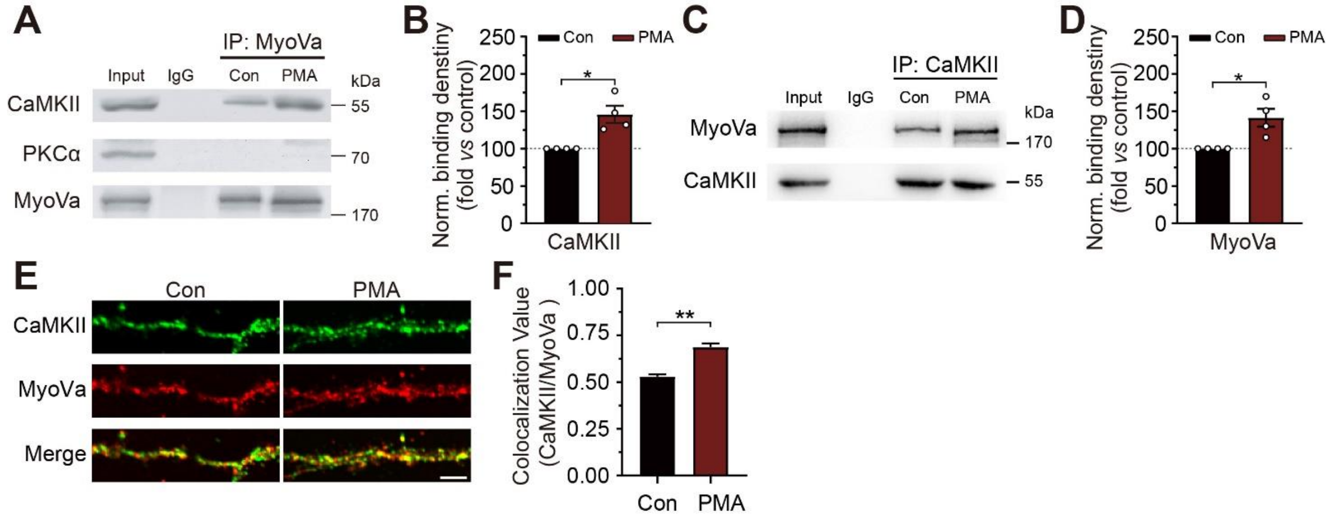

Fig. S5 The association of MyoVa and CaMKII is enhanced during PMA-induced NMDAR transport. **A–D** Co-IP assays indicate that the binding between MyoVa and CaMKII is strengthened under PMA conditions. In contrast, PKCα does not bind to MyoVa. The association between MyoVa and CaMKII was examined using antibodies against MyoVa (**A–B**) or CaMKII (**C–D**). Non-immune IgG was used as control. Signals were divided by MyoVa (**B**) or CaMKII (**D**) signals and normalized to control. For IP with MyoVa: n = 4, the binding is increased to 1.46 ± 0.11, P <0.05; for IP with CaMKII: n = 4, 1.42 ± 0.12, P <0.05; unpaired Student's t-test *vs* control. **E** Immunofluorescence assays indicating that PMA treatment significantly increases the colocalization of CaMKII and MyoVa. Primary hippocampal neurons are stained with CaMKII (green) and MyoVa (red) antibodies in control or under PMA conditions. The data are from at least three independent cultures. Scale bar, 5 μm. **F** Quantification of the colocalization between CaMKII and MyoVa by Pearson's coefficient. Control: n = 21, 0.53 ± 0.01; PMA, n = 19, 0.69 ± 0.02, P <0.01; unpaired Student's t-test *vs* control. The data are represented as the mean ± SEM, $^{*}P$ <0.05; $^{**}P$ <0.01.

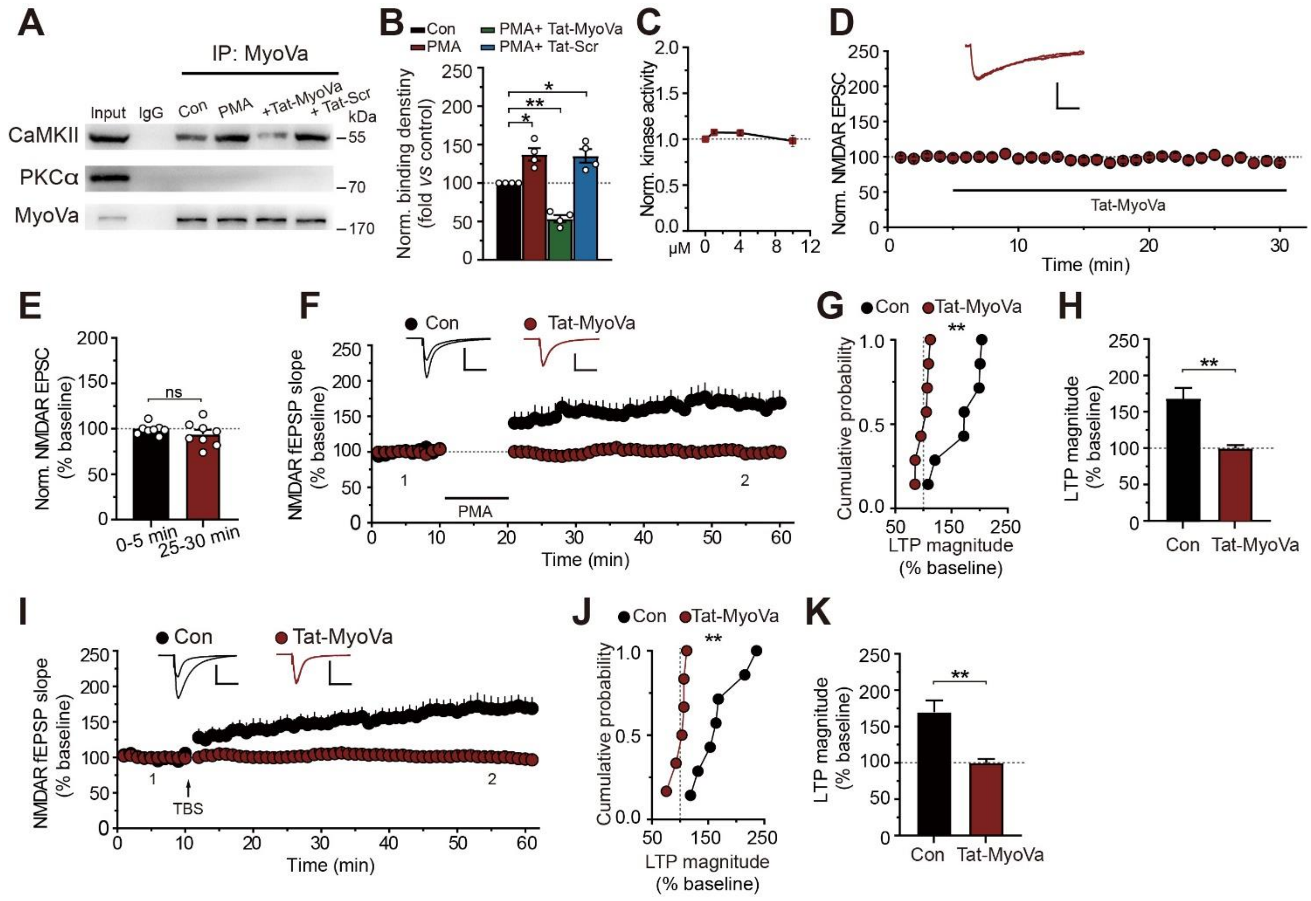

Fig. S6 The effect of Tat-MyoVa on CaMKII-MyoVa interaction, CaMKII activity, and NMDAR-mediated synaptic currents. **A** Co-IP assay reveals that the short cell membrane-permeable interfering peptides Tat-MyoVa (4 μmol/L) reduce the association of MyoVa with CaMKII under PMA conditions. Comparatively, the scrambled peptides Tat-Scr do not influence the association. **B** Statistical analysis of the association level between MyoVa and CaMKII. The CaMKII band density was divided by the MyoVa band density and subsequently normalized to the control condition. PMA, 1.37 ± 0.08, P <0.05; PMA + Tat-MyoVa, 0.54 ± 0.05, P <0.01; PMA + Tat-Scr, 1.35 ± 0.09, P <0.05; n = 4; one-way repeated-measures ANOVA *vs* control. **C** No significant change in normalized CaMKII activity under Tat-MyoVa treatment. n = 4, P >0.05; one-way repeated-measures ANOVA. **D** No significant change in normalized evoked NMDA EPSCs after Tat-MyoVa treatment. **E** Normalized NMDAR EPSCs before and after Tat-MyoVa treatment. Calculated from the data in C. n = 8, P >0.05, unpaired Student's t-test. Scale bars, 25 pA, 100 ms in **D**. **F–H** Tat-MyoVa blocks PMA-induced LTP of NMDA fEPSPs. The overlaid traces display changes in the average response selected at the times shown (marked by 1 and 2). **G** Cumulative probability of potentiation magnitude of NMDA fEPSPs. **H** Summary graphs of LTP magnitude from experiments shown in **F**. Control: 1.68 ± 0.15, n = 7; Tat-MyoVa: 1.00 ± 0.04, n

= 7; *P* <0.01; unpaired Student's *t*-test *vs* control. Scale bars, 0.5 mV, 100 ms in **F**. **I–K** As in **F–H**, with the exception that LTP of NMDA fEPSPs was induced by TBS. Control: 1.69 ± 0.16, *n* = 7; Tat-MyoVa: 1.00 ± 0.05, *n* = 6, *P* <0.01; unpaired Student's *t*-test *vs* control. Scale bars, 0.5 mV, 100 ms in **I**. The data are represented as the mean ± SEM, $^{*}P$ <0.05; $^{**}P$ <0.01; ns, no significant difference.

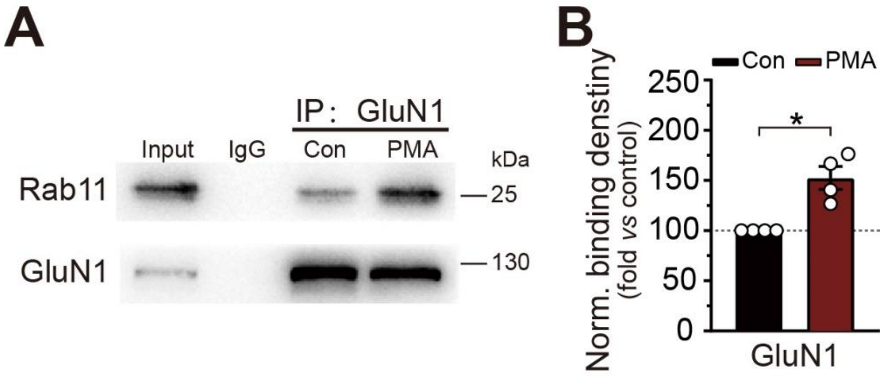

Fig. S7 The association of Rab11-GluN1 is enhanced during PMA-induced NMDAR transport. **A** Co-IP experiments with antibodies to GluN1 reveal increased GluN1-Rab11 association. Non-immune IgG was used as controls. **B** Statistical analysis of binding from experiments in **A**. All signals were divided by GluN1 signals and normalized to control. The binding is increased to 1.53 ± 0.11, *P* <0.05, *n* = 4; unpaired Student's *t*-test *vs* control. The data are represented as the mean ± SEM, $^{*}P$ <0.05.

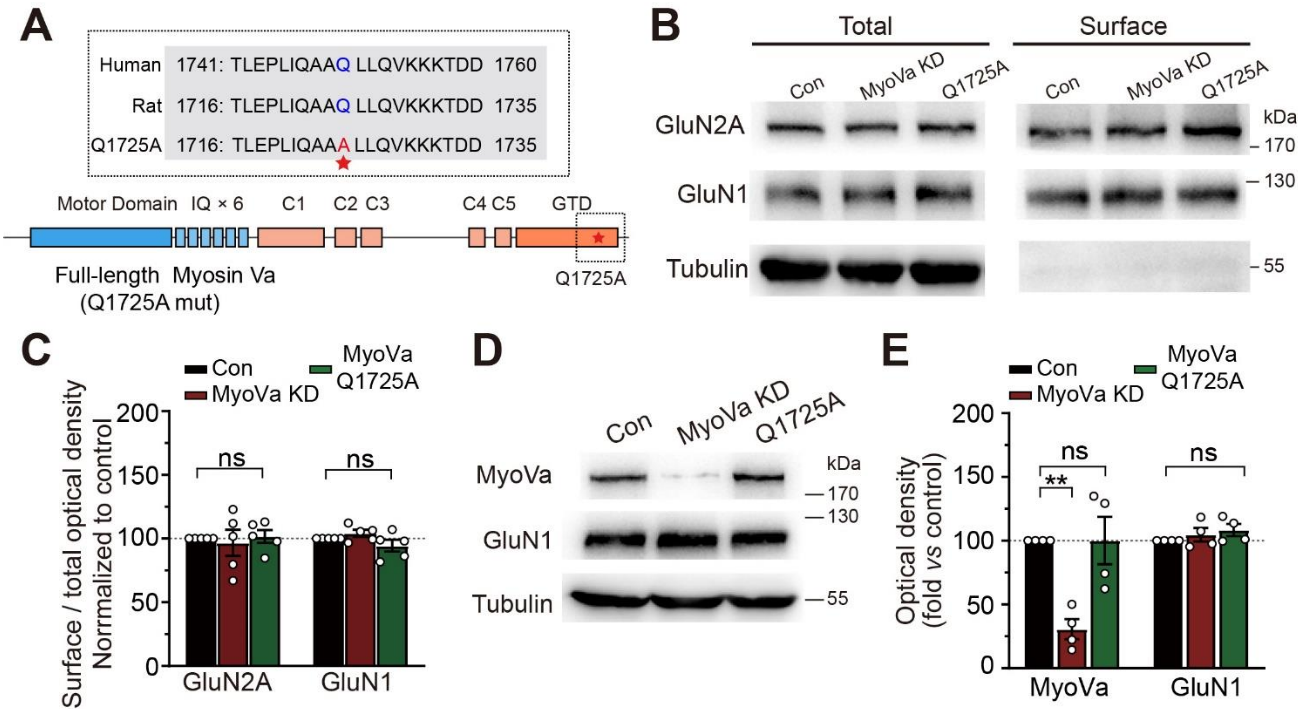

Fig. S8 The expression of Q1725A mutant MyoVa in hippocampal neurons. **A** Upper panel, the alignment of a segment of sequence in the MyoVa GTD. The 1750 site in humans, which is critical for its interaction with Rab11, is homologous to that of 1725 in rats. Lower panel, schematic diagram of the full-length Q1725A mutant MyoVa (Q1725A mut) used in Fig. 6. **B** The expression of GluN1 and GluN2A on the surface is unaffected under basal condition in MyoVa KD and Q1725A neurons. Western blots comparing surface and total NMDAR subunit expression under different experimental treatments. **C** NMDAR subunit surface/total protein ratios shown in **B**. Data represent band intensity relative to control values. GluN1: MyoVa KD, 1.04 ± 0.03, *P* >0.05; Q1725A, 0.94 ± 0.05, *P* >0.05; GluN2A: MyoVa KD, 0.97 ± 0.10, *P* >0.05; Q1725A, 1.02 ± 0.05, *P* >0.05; *n* = 5; one-way repeated-measures ANOVA *vs* control. **D** Western blots verifying MyoVa and GluN1 levels in neurons expressing Q1725A mutant MyoVa. The expression levels of MyoVa and GluN1 are comparable in the control and Q1725A mutant. **E** Quantification of the results in **D**. Q1725A mutant MyoVa shows MyoVa and GluN1 expression levels comparable to control. Data represent band intensity relative to control. MyoVa: KD, 0.31 ± 0.08, *P* <0.01; Q1725A, 1.00 ± 0.19, *P* >0.05; GluN1: KD, 1.05 ± 0.05, *P* >0.05; Q1725A, 1.08 ± 0.05, *P* >0.05; *n* = 4; one-way repeated-measures ANOVA *vs* control. The data are represented as the mean ± SEM, ***P* <0.01. ns, no significant difference.

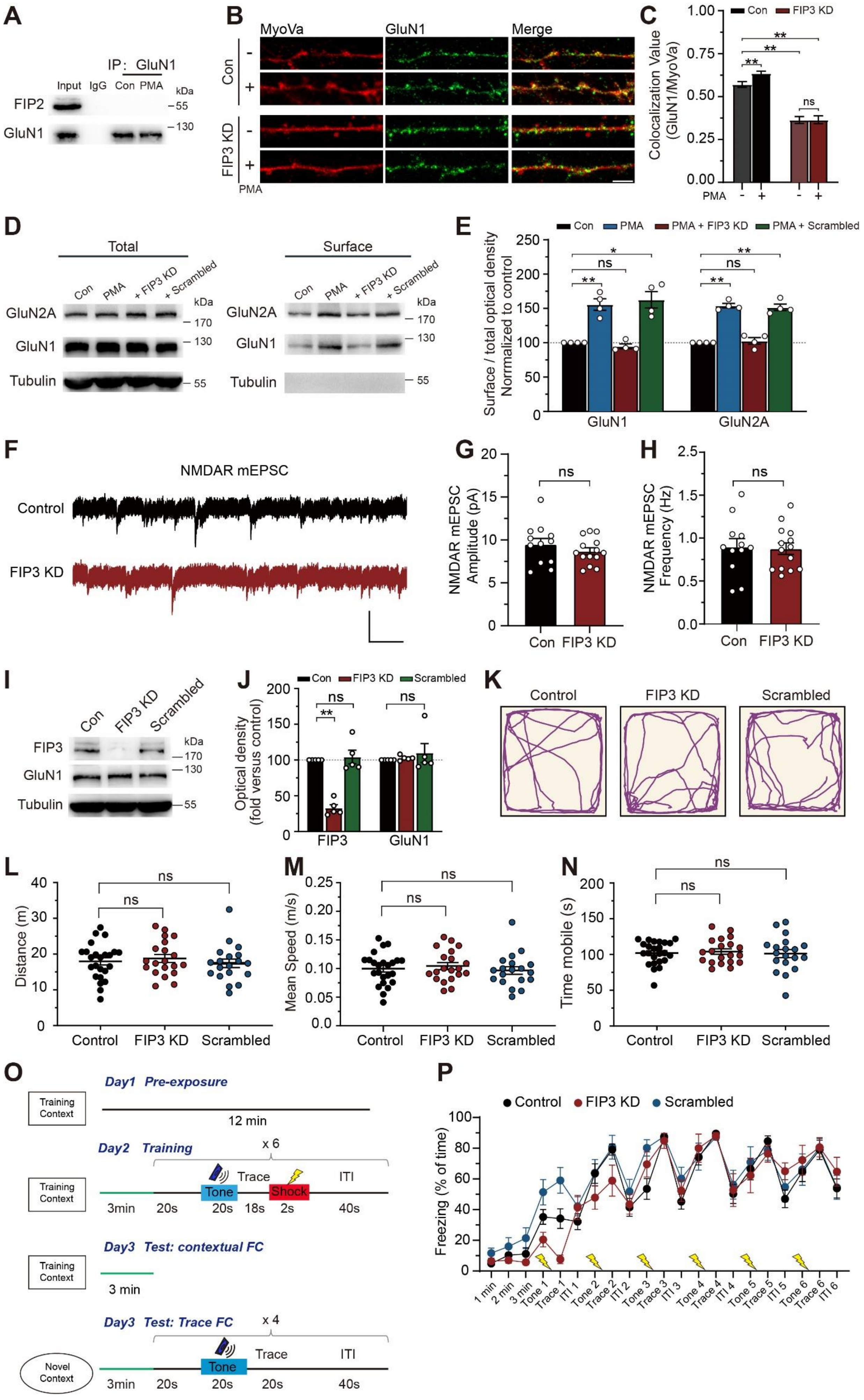
A
IP: GluN1
Input IgG Con PMA
kDa
FIP2 55
GluN1 130
B
MyoVa GluN1 Merge
Con - +
FIP3 KD - +
PMA
C
Con FIP3 KD
Colocalization Value (GluN1/MyoVa)
PMA - + - +
ns
D
Total
Surface
Con PMA + FIP3 KD + Scrambled
GluN2A 170
GluN1 130
Tubulin 55
E
Con PMA PMA + FIP3 KD PMA + Scrambled
Surface / total optical density Normalized to control
GluN1 GluN2A
ns
F
NMDAR mEPSC
Control
FIP3 KD
G
NMDAR mEPSC Amplitude (pA)
Con FIP3 KD
ns
H
NMDAR mEPSC Frequency (Hz)
Con FIP3 KD
ns
I
Con FIP3 KD Scrambled
FIP3 GluN1 Tubulin
J
Con FIP3 KD Scrambled
Optical density (fold versus control)
FIP3 GluN1
K
Control FIP3 KD Scrambled
L
Distance (m)
Control FIP3 KD Scrambled
M
Mean Speed (m/s)
Control FIP3 KD Scrambled
N
Time mobile (s)
Control FIP3 KD Scrambled
O
Day1 Pre-exposure
Training Context
12 min
Day2 Training
x 6
Tone Trace Shock ITI
3min 20s 20s 18s 2s 40s
Day3 Test: contextual FC
3 min
Day3 Test: Trace FC
x 4
Novel Context
Tone Trace ITI
3min 20s 20s 20s 40s
P
Control FIP3 KD Scrambled
Freezing (% of time)
1 min 2 min 3 min Tone 1 Trace 1 ITI 1 Tone 2 Trace 2 ITI 2 Tone 3 Trace 3 ITI 3 Tone 4 Trace 4 ITI 4 Tone 5 Trace 5 ITI 5 Tone 6 Trace 6 ITI 6

Fig. S9 The effect of FIP3 KD on protein expression, NMDAR mEPSCs, and animal behavior. **A** Co-IP experiments with antibodies against GluN1 reveal that GluN1 does not bind to FIP2. Non-immune IgG was used as a control. The experiment was performed independently at least 3 times. **B** Immunofluorescence assays reveal that the colocalization between MyoVa (red) and GluN1 (green) is significantly reduced in FIP3 KD neurons. Scale bar, 5 μm. **C** Quantification of the colocalization between GluN1 and MyoVa by Pearson's coefficient. Control: n = 24 neurons, 0.57 ± 0.02; PMA: n = 24 neurons, 0.63 ± 0.01, P <0.01; FIP3 KD: n = 27 neurons, 0.36 ± 0.02, P <0.01; FIP3 KD-PMA: n = 27 neurons, 0.36 ± 0.02, P <0.01; data are from at least three independent cultures; one-way repeated-measures ANOVA *vs* control. **D** The expression of GluN1 and GluN2A on the surface is impaired in FIP3 KD neurons. Western blots comparing surface and total NMDAR subunit expression under different experimental treatments. **E** The surface/total ratio of NMDAR subunits shown in **D**. Data represent band intensity relative to control values. GluN1: PMA, 1.56 ± 0.08, P <0.01; FIP3 KD, 0.95 ± 0.04, P >0.05; scrambled, 1.63 ± 0.12, P <0.05; GluN2A: PMA, 1.54 ± 0.03, P <0.01; FIP3 KD, 1.03 ± 0.05, P >0.05; scrambled, 1.52 ± 0.05, P <0.01; n = 4; one-way repeated-measures ANOVA *vs* control. **F–H** FIP3 KD does not affect the amplitude and frequency of NMDAR mEPSCs. Representative mEPSC traces are shown in **F**; mEPSC amplitude and frequency are shown in **G** and **H**, respectively. Amplitude (**G**): control: n = 12, 9.49 ± 0.69; MyoVa KD: n = 14, 8.68 ± 0.41, P >0.05. Frequency (**H**): control: n = 12, 0.90 ± 0.09; MyoVa KD: n = 14, 0.88 ± 0.07, P >0.05; unpaired Student's t-test *vs* control. Scale bars, 10 pA, 2s in **F.** **I** Western blots verifying FIP3 and GluN1 expression levels in slices infected with FIP3 knockdown or scrambled virus. FIP3 rather than GluN1 is markedly reduced in FIP3 KD but not in scrambled. **J** The protein expression levels of FIP3 and GluN1. Data represent band intensity relative to control. FIP3: KD, 0.33 ± 0.05, P <0.01; scrambled, 1.04 ± 0.09, P >0.05; GluN1: KD, 1.03 ± 0.02, P >0.05; scrambled, 1.10 ± 0.13, P >0.05; n = 5; one-way repeated-measures ANOVA *vs* control. **K–N** Expression of FIP3 knockdown or scrambled viruses have no effect on locomotor behaviors. Control: n = 24; KD: n = 20; scrambled: n = 19; P >0.05, one-way repeated-measures ANOVA *vs* control. **O** Schematic of the fear conditioning paradigm protocol. **P** Animals expressing FIP3 knockdown or scrambled viruses exhibit freezing levels comparable to control and scrambled animals. Two-way ANOVA, P >0.05. The data are represented as the mean ± SEM, $^{*}P$ <0.05; $^{**}P$ <0.01; ns, no significant difference.

A

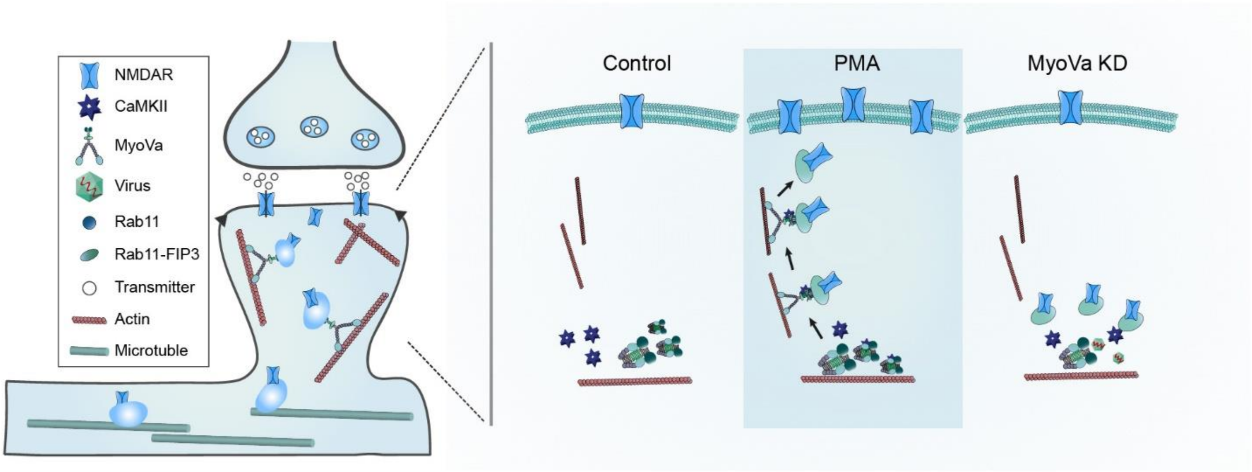

Fig. S10 Diagram of the putative process of MyoVa-dependent transport of NMDARs in response to activity inputs in hippocampal neurons. **A** Once PKC is activated by PMA, more NMDAR channels are opened, allowing Ca^{2+} influx through them. At the base of the dendritic spine, a local Ca^{2+} increase in response to activity inputs activates CaMKII. The activated CaMKII strengthens its direct association with MyoVa, which in turn triggers a conformational switch of MyoVa that exposes the globular tail domain (GTD), possibly *via* phosphorylation of the neighboring consensus CaMKII phosphorylation site. This MyoVa activation facilitates a stronger association between MyoVa and NMDARs for subsequent NMDAR transport. In the transportation process, Rab11/FIP3 act as the adaptor proteins to couple NMDARs and MyoVa (left shadowed region of enlarged diagram). MyoVa KD or interference with the association of CaMKII with MyoVa jeopardizes the NMDAR delivery to the postsynaptic membrane (right region of the enlarged diagram). Arrows connect crucial binding stages that are involved in NMDAR surface transport.
